# What’s the risk? Identifying potential human pathogens within grey-headed flying foxes faeces

**DOI:** 10.1371/journal.pone.0191301

**Published:** 2018-01-23

**Authors:** Rebekah Henry, Penelope Galbraith, Scott Coutts, Toby Prosser, John Boyce, David T. McCarthy

**Affiliations:** 1 Environmental and Public Health Microbiology Laboratory (EPHM Lab), Department of Civil Engineering, Monash University, Clayton, Victoria, Australia; 2 Micromon, Dept. of Microbiology, Monash University, Clayton, Victoria, Australia; 3 Melbourne Water, Docklands, Victoria, Australia; 4 Department of Microbiology, Monash University, Clayton, Victoria, Australia; CSIRO, AUSTRALIA

## Abstract

*Pteropus poliocephalus* (grey-headed flying foxes) are recognised vectors for a range of potentially fatal human pathogens. However, to date research has primarily focused on viral disease carriage, overlooking bacterial pathogens, which also represent a significant human disease risk. The current study applied 16S rRNA amplicon sequencing, community analysis and a multi-tiered database OTU picking approach to identify faecal-derived zoonotic bacteria within two colonies of *P*. *poliocephalus* from Victoria, Australia. Our data show that sequences associated with *Enterobacteriaceae* (62.8% ± 24.7%), *Pasteurellaceae* (19.9% ± 25.7%) and *Moraxellaceae* (9.4% ± 11.8%) dominate flying fox faeces. Further colony specific differences in bacterial faecal colonisation patterns were also identified. In total, 34 potential pathogens, representing 15 genera, were identified. However, species level definition was only possible for *Clostridium perfringens*, which likely represents a low infectious risk due to the low proportion observed within the faeces and high infectious dose required for transmission. In contrast, sequences associated with other pathogenic species clusters such as *Haemophilus haemolyticus-H*. *influenzae* and *Salmonella bongori-S*. *enterica*, were present at high proportions in the faeces, and due to their relatively low infectious doses and modes of transmissions, represent a greater potential human disease risk. These analyses of the microbial community composition of *Pteropus poliocephalus* have significantly advanced our understanding of the potential bacterial disease risk associated with flying foxes and should direct future epidemiological and quantitative microbial risk assessments to further define the health risks presented by these animals.

## Introduction

Bats are among the most species-rich mammals on earth and provide many essential ecological services including seed dispersion, pollination and arthropod suppression [1]. Further, bats are one of the most abundant mammalian species on the planet due to their ability to form large aggregated colonies and, for some orders, adapt to and thrive under increasing urban pressures [2]. Consequently, they are animals of key economic and environmental significance [3]. Traditionally there have been two recognised sub-orders of bats; Microchiroptera (order Chiroptera) i.e. “microbats” and Megachiroptera i.e. “megabats”. In this classification system, Megachiroptera comprise one family, *Pteropodidae*, encompassing all species of flying fox (collectively referred to as Pteropid bats) [4]. However, recent taxonomic evaluation, based on genomic and transcriptomic information, has resulted in the re-classification of Pteropid bats within Yinpterochiroptera (or Pteropodiformes); which includes the taxa formerly known as megabats as well as some microbat taxa [5].

Pteropid bats have been shown to carry a number of significant viral pathogens including Ebola [6], Hendra [7], Nipah [8], Menangle, Tioman, Australian Bat Lyssavirus and novel members of the Rubulavirus genus [9]. These diseases can be transmitted via a number of pathways including, bites, scratches and contact with secretions from infected animals, although for some disease such as Hendrah and Nipah an intermediate amplification host is usually required. Despite growing evidence elucidating the role of Pteropids in zoonotic disease transmission, the risks of indirect pathogen transmission by bats, predominantly associated with contact with deposited faeces, remain poorly characterised [10, 11]. The close association of bat colonies with highly urbanised environments represents a possibly significant health risk, with faecal deposits the likely first contact point between human and bat populations [12]. However, rooftop deposition and increasing public use of rainwater tanks means that indirect disease transmission, through consumption and contact with contaminated water supplies and/or areas irrigated by these supplies can also occur.

Numerous studies have attempted to describe the normal gastrointestinal (GI) flora of bats. However, results to date provide an incomplete snapshot of both the total and pathogenic bacterial communities in bat GI environments. Despite this, studies have demonstrated that bacteria from the families *Enterobacteriaceae* and *Staphylococcaceae* predominate in the Pteropid gastrointestinal tract. Bacterial pathogens including, *Listeria monocytogenes* [13], *Shigella sonnei* [14], *Leptospira* spp. [15] and several species of *Salmonella* [14, 16, 17] have also been identified within the kidney and GI tract of some species. A small number of studies have focused on characterisation of pathogens from bat faeces with *Salmonella*, *Coxiella* and *Pseudomonas* spp. having been reported [18, 19].

Next-generation sequencing (NGS) methods can be used to directly analyse bacterial microflora using DNA extracted from animal faecal deposits [20, 21]. These direct sequencing methods overcome many of the limitations of traditional culture-based methods and allow for a more comprehensive characterisation of entire bacterial populations (including non-culturable species) [22]. Consequently, several metagenomics-based NGS studies have profiled the microbial community composition of *Microchiropteran* bat faeces [11, 23, 24]. However, to date, no studies have characterised the bacterial faecal microbiome of Pteropid bats, despite their clearly recognised role as human-disease vectors.

The current study employed NGS 16S rRNA amplicon sequencing to define the bacterial community composition of Pteropid (*Pteropus poliocephalus*) faeces. The potential role of bats as a vector for zoonotic disease was directly investigated *via* identification of unique sequences associated with previously characterised human pathogen taxa. The study aimed to apply these findings to enhance our limited understanding of the bacterial communities and human-transmissible bacterial pathogens associated with flying foxes. This research will enable future quantitative microbial risk assessments to be undertaken into the human health risks presented by these species within urban environments.

## Materials and methods

### Faecal sample collection

Flying fox faecal samples (n = 18) were collected between 17/04/14 and 11/8/14 from two urban flying fox colonies located at Doveton (n = 5, GPS: -37.977333, 145.235361) and Yarra Bend (n = 13, GPS: -37.796229, 145.018000), Melbourne, Australia. Faeces were collected after natural defecation by the animals into the environment, in order to minimise interaction times and stress to the flying fox colony. Bat faeces was collected from a wooden pier located underneath the bat colony, which was cleaned of old faeces after each collection. Deposits which were made on this structure, in the presence of the investigator, were collected and processed. The total faecal deposit was then collected for further analysis. Faeces from other animal sources (n = 68, 17/04/14-11/8/14) were additionally collected, post natural defecation to prevent undue stress, for cross-comparative bacterial community analysis (S1 File). Freshly deposited material was collected in sterile plastic containers and transported at 4°C to the Environmental and Public Health Microbiology laboratory (Monash University, Clayton) within 2–4 h of collection [25]. The samples were manually homogenised, weighed, and 0.25 g faeces sub-samples were stored at -20°C prior to genomic DNA extraction. The total moisture content was determined from 2 g of the remaining homogenised material following the method described in Australian Standard AS 1289.2.1.1–2005 [26].

### Total genomic DNA isolation

Faecal sub-samples (0.25 g) were thawed and then genomic DNA extracted using the PowerSoil^®^ DNA Isolation Kit (MoBio Laboratories Inc., Carlsbad, CA) as per manufacturer instructions, with the following modifications. Prior to extraction, sub-samples were re-weighed to ensure accurate quantification. DNA was eluted in a final volume of 50 μL sterile RNA/DNA free water. DNA yields in final eluates were quantified using a Qubit fluorometric system (Thermo Fisher Scientific, Canada). All extracts were stored at -20°C prior to 16S amplicon sequencing.

### Library preparation and 16S amplicon sequencing

Illumina library preparation and 16S amplicon sequencing were conducted as previously described [25]. Briefly, the V3-4 region of the bacterial rRNA gene was amplified in triplicate for each sample using 50 μL PCR reactions containing: 5 μL of genomic DNA, 1x PCR buffer (Roche), 0.3 μL of Taq polymerase (Roche), 1 μM of forward (5’-TCGTCGGCAGCGTCAGATGTGTATAAGAGACAGCCTACGGGNGGCWGCAG) and reverse (5’- GTCTCGTGGGCTCGGAGATGTGTATAAGAGACAGGACTACHVGGGTATCTAATCC) primer. Reactions underwent initial denaturation at 98°C followed by 25 cycles of denaturation at 98°C, annealing at 55°C and extension at 72°C, each for 30 sec. Final extension was conducted for 5 min at 72°C. PCR products were subsequently purified using 0.6 volumes of Ampure XP (Beckman-Coulter), as per manufacturer’s instructions. Triplicate 5 μL sub-sample of the purified PCR product was used as the template for secondary PCR amplification to add Illumina-compatible sequencing adapters and unique per-sample indexes using the Nextera XT Indexing Kit (Illumina). The DNA from each triplicate reaction was pooled and purified using 0.6 volumes of Ampure XP. The resulting amplicon mix was quantified for each sample using the Qubit and Qubit dsDNA HS Kit (Invitrogen), normalised, pooled and sequenced using a MiSeq V3 600c Reagent Kit (Illumina).

### Quality filtering and OTU picking

Quality filtering and OTU picking procedures have been described elsewhere [25, 27]. Briefly, sequencing data were demultiplexed using MiSeq Reporter V2.4.60, filtered to remove adapters and trimmed to remove any terminal stretches of bases at or below Q30 using Trimmomatic [28]. Reads shorter than 180 nt were discarded. Pre-cluster read pairs were trimmed and filtered to produce single reads using PEAR [29], requiring a minimum 50 bp overlap. The assembled reads were analysed using the QIIME 1.8.0 closed-reference OTU picking workflow with UCLUST for *de novo* OTU picking and the GreenGenes 13_8 release for the reference and for taxonomic identity assignment [30]. Prior to analysis, all singleton and doubleton sequences were removed through application of the filter_otus_from_otu_table.py script passing -n 2 and -n 3 to increase precision of OTU characterisation in downstream analyses [31]. Data for all samples are available on the Short Read Archive, project reference PRJNA309092 (https://www.ncbi.nlm.nih.gov/bioproject/PRJNA309092/).

### Diversity analysis

Microbial diversity was evaluated within samples (α-diversity) and between samples (β-diversity) using QIIME [30]. All scripts are presented in S2 File. Rarefaction to a sub-sampling depth of 40,000 reads/sample was performed on all flying fox faeces samples for diversity analysis. Alpha-diversity was measured using, observed species, Shannon diversity (OTU-based diversity) [32], Simpson diversity and Simpson Equitability index (evenness) metrics. Beta diversity was measured with weighted (quantitative measure of species abundance) and unweighted (qualitative measure of species presence/absence) UniFrac analysis as the use of both measures provides different insights into community relationships (Lozupone et al., 2007). Unweighted and weighted UniFrac-based Principal Co-ordinate Analysis (PCoA) was conducted to characterise the dissimilarity between the bacterial communities within each faecal sample type. Sample means and standard error of the mean (SEM) were calculated using GraphPad PRISM software (GraphPad Software, CA, USA).

Comparative α- and β-diversity analysis was conducted between *Pteropus poliocephalus* faeces and that of 14 other animals found in the same geographic location (Melbourne Australia). These were: human (n = 10), possum (ring-tailed and brush-tailed possum) (n = 2), sheep (n = 2), horse (n = 5), rabbit (n = 3), dog (n = 5), cat (n = 5), birds (common myna, silver gull, rock dove) (n = 4), cow (n = 8), pig (n = 6), chicken (n = 5), goat (n = 2) and waterfowl (Eurasian coot, purple swamphen and Australian wood duck) (n = 11). Microbial diversity was assessed using the core_diversity.py analysis script and the α- and β-diversity metrics described above. For inter-species comparison, rarefaction to a sub-sampling depth of 20,000 reads/sample was performed on closed-OTU tables prior to diversity analysis.

### Identification of faecal associated zoonotic pathogens

The ability to accurately define zoonotic pathogens associated with flying fox populations is dependent on the tools and databases used for data analysis. As GreenGenes 13_8 has a recognised dearth of species level sequence information [33], a second round of taxonomic classification was conducted. Further species level classification and phylogenetic analysis was performed to increase confidence in these results. These processes are described in the following sections.

#### Genbank species classification at 97% clustering

For this secondary analysis, the assembled reads were analysed using the QIIME 1.8.0 closed-reference OTU picking workflow with UCLUST for *de novo* OTU picking (as described above) and the Genbank reference database (access date 02/09/2016) for assignment of taxonomic identity [30]. Clustering at 97% was maintained for this analysis to allow comparison with previously annotated data. For Genbank analysis, default parameters were applied to parallel_pick_otus_blast.py (97% similarity, E = 1e-10, minimum aligned percent = 0.5). OTUs containing <10 sequences in any of the Pteropid faecal samples were removed from further analysis.

#### Identification of human pathogens within Genbank dataset

A total of 639 bacterial species were identified. To specifically identify pathogenic bacteria a literature survey was conducted using Google Scholar (https://scholar.google.com.au) and NCBI PubMed (https://www.ncbi.nlm.nih.gov/). Each identified species was researched and designated as either a human pathogen (i.e. having caused reported human disease) or non-pathogen.

Human pathogens were further separated into: 1) those primarily causing opportunistic/hospital-acquired infections and 2) those primarily associated with community-acquired and/or food-borne illness (S1 File). Bacteria classified into category 2, and their associated reads underwent further analysis.

#### Confirmation of species level classification

The raw sequence reads for each remaining OTU were filtered from the raw FASTA file using the q2oligo.py and filter_fasta.py scripts. To improve classification confidence, filtered reads were then applied to the sequence search tool, search & classify function of SILVA (//www.arb-silva.de/aligner/) [34]. Matches were made against the Ribosomal Database Project (RDP)[35], GreenGenes and SILVA databases. A positive classification was considered if >90% identity over the length of the ~450bp amplicon was achieved to the same genus and species (where available) as was obtained by Genbank. Bacteria were removed from further investigation if all three tools were unable to classify below family level. To confirm putative pathogen identity, the remaining raw sequence reads were matched against the Genbank nucleotide database using BLAST [29] under default conditions (Expect 10, word size 28).

#### Preparation of reference amplicons

Reference sequences for each shortlisted bacterial genus were obtained from the SILVA database Version 128 [34]. SILVA TestPrime (www.arb-silva.de/search/testprime/) was applied to ensure data was obtained only for species (within each specified genus) that could be amplified by the specified V3-V4 rRNA oligonucleotides. The results were then filtered to remove data associated with all unknown, uncultured or uncharacterised sequences as well as those belonging to other species or genera incorrectly classified. *In silico* PCR was conducted using IPCRESS (http://www.ebi.ac.uk/about/vertebrate-genomics/software/) to produce reference amplicons for comparative alignment and analysis.

#### Identity matrix analysis

To define the within-genus sequence identity across the ~ 500 bp region, reference amplicons were aligned using Clustal Omega [36] under default parameters. Concurrently, the Percent Identity Matrix (PIM) values were calculated for each species within the investigated genera (S4 File). Identity matrix values of ≥97% indicated species which could not be accurately defined to this phylogenetic level using the QIIME clustering threshold set for this study.

#### Phylogenetic analysis

The filtered reads of remaining bacteria were combined with the previously prepared reference amplicons and aligned using MUSCLE [37] v3.8.31 under default conditions. To assess inferred relationships, Maximum-Likelihood phylogenetic trees were constructed based on the Tamura-Nei model [38] using MEGA7 [39] (S3 File). Further, confirmation of taxonomic assignment was conducted using Taxonomer on the remaining filtered reads [40].

## Results

*Next generation sequence analysis of bat faecal samples*. A total of 1, 817, 323 sequence reads were generated for the 18 faecal samples. The application of closed reference OTU picking and GreenGenes taxonomic classification identified 32, 324 unique taxa accounting for 1, 077, 879 (59.3%; samples mean ± SD of 59882 ± 21441) of the total sequence reads. Of the total observed OTUs, 94.3% were singletons and a further 20.1% were doubletons. Genomic DNA from bat faeces sample 9 (Bat-9) generated only 1, 452 sequences; significantly below the average yield of the other samples and was consequently removed from further investigation.

**Fig 1:**
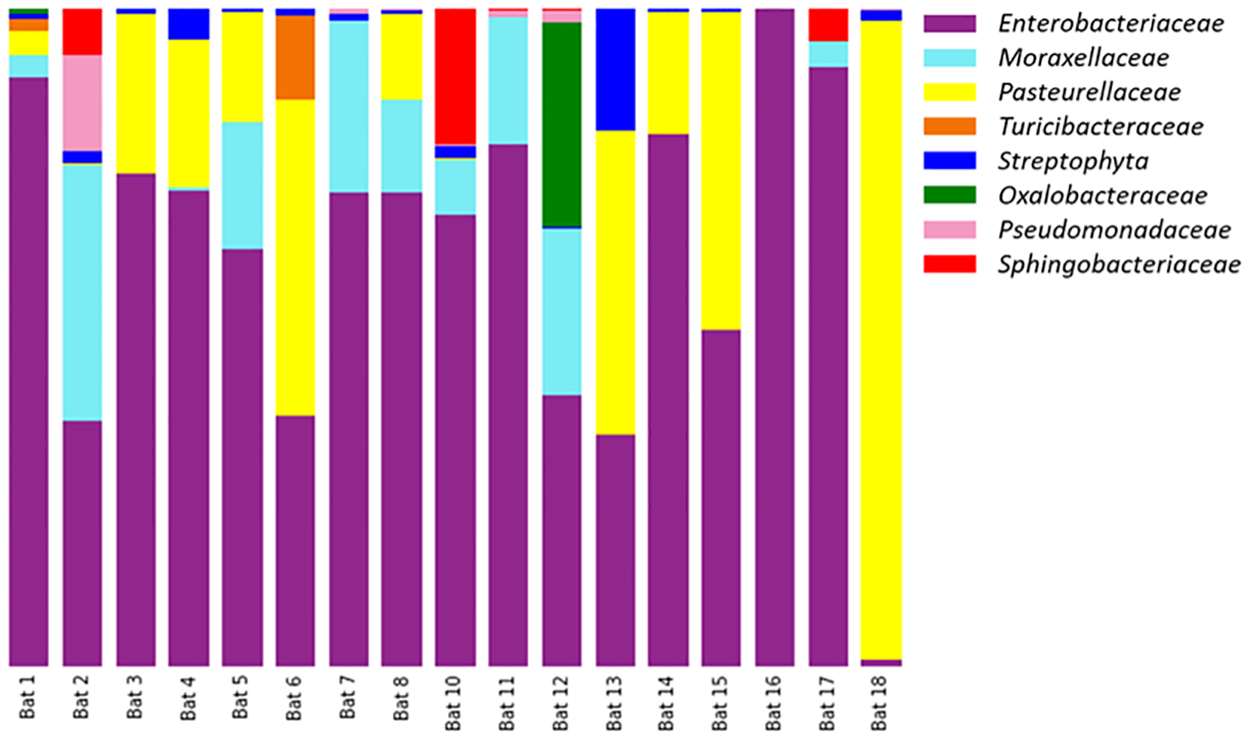
Proportion of most abundant taxa with mean relative abundances of bacterial families with >0.5% total sequence reads. Bat faeces 1–13 collected from Yarra Bend (Victoria, Melbourne). Bat faeces 14–18 collected from Doveton (Victoria, Australia).

*Bacterial community profile of* Pteropus poliocephalus *faeces*. Taxonomic profiles of the genera present within the faecal samples consisted primarily of a small number of bacterial families. In total, seven bacterial families (and one archaea) were observed to have mean relative sequence abundances of >0.5% across the 17 *Pteropus poliocephalus* samples (Fig 1). However, on average the flying fox faeces were dominated by sequences associated with *Enterobacteriaceae* (62.8% ± 24.7%), *Pasteurellaceae* (19.9% ± 25.7%) and *Moraxellaceae* (9.4% ± 11.8%).

*Microbial diversity within and between* Pteropus poliocephalu*s colonies*. Analysis of α-diversity metrics was conducted to compare the faecal microbiome from the Yarra Bend and Doveton bat colonies (Table 1). The two populations showed significant differences in all selected ecological measures. For metrics including observed species, Shannon and Simpson indices, a greater average diversity was observed in the faecal microbiome from the Yarra Bend colony compared to the Doveton colony microbiome (449 vs. 228, 4.26 vs. 2.84 and 0.87 vs. 0.76, respectively).

**Table 1:** Indices for the diversity, evenness and richness between Doveton and Yarra Bend (mean ± standard deviation) bat colonies faecal microbiome.

Indices	Doveton (Mean)	Doveton (Std Dev)	Yarra Bend (Mean)	Yarra Bend (Std Dev)	P-value^b^
**Observed species**	228.04	139.00	448.98	191.28	0.044
**Shannon**	2.84	0.42	4.26	0.95	0.011
**Simpson**	0.76	0.06	0.87	0.06	0.006
**Evenness**^a^	0.008	0.004	0.003	0.003	0.023

^a^Evenness of each sample as calculated by Simpson Equitability index

^b^P-value derived from two-sample t-test comparison of alpha diversity indices at rarefaction depth of 40000 sequences.

**Fig 2:**
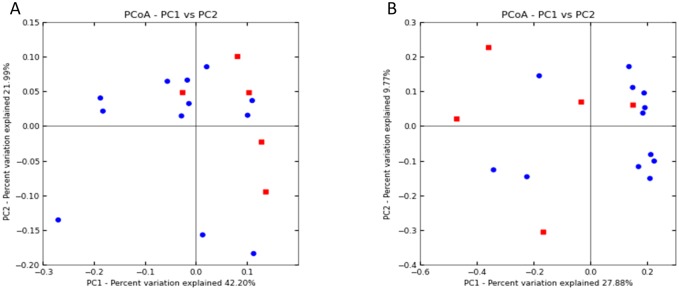
Principal co-ordinate analysis (PCoA) of the identified microbiomes of Yarra Bend (blue) and Doveton (red) *Pteropus poliocephalus* faeces. Plots were generated using A) weighted and B) unweighted UniFrac distance matrices.

OTUs that were common to all *Pteropus poliocephalus* samples or specific to samples from either collection site were identified. All analysed *Pteropus poliocephalus* samples comprised sequences from species within the genus *Actinobacillus* and the family *Enterobacteriaceae*. Amplicons belonging to order Archaea were also identified within all samples and are likely associated with the consumption of a common terrestrial food source. The bacterial genera *Enterobacter*, *Erwina*, *Acinetobacter* and *Pseudomonas* were identified in all Yarra Bend samples. In contrast, *Haemophilus* sequences and amplicons associated with the family *Clostridiaceae* were observed in all Doveton scats. Unweighted and weighted UniFrac metrics were applied in β-diversity analysis to further define associations between Yarra Bend and Doveton colonies. Principal co-ordinate analysis (PCoA) of unweighted Unifrac distances revealed significant clustering based on location (ANOSIM and PERMANOVA; P <0.05) (Fig 2). However, the same relationship was not observed in weighted Unifrac analysis.

*Microbial diversity between* Pteropus poliocephalu*s and local animal populations*. Comparative analysis of α-diversity metrics was conducted to compare the faecal microbiome from the Yarra Bend and Doveton bat colonies with that of regionally relevant animal populations (Table 2). Significant differences (P <0.05) were observed between agriculturally-derived faeces (cow, goat, pig and sheep) and that of the fruit bats under all selected ecological measures, except for Simpson Equitability index. Higher diversity was observed for cow, goat and sheep samples (Table 2). It is important to note that the rarefaction curves for these animals did not reach a plateau, suggesting that a rarefaction depth greater than the applied 20,000 sequences is required to completely characterise the microbial community. For all remaining animals, a plateau was reached in observed species and Chao1 indices.

**Table 2:** Average indices for the richness, diversity and evenness between the faecal microbiomes of the combined bat colonies and that of regionally specific animal species.

Indices^a^	Bat (n = 18)	Bird (n = 4)	Cat (n = 5)	Chicken (n = 5)	Cow (n = 8)	Dog (n = 5)	Goat (n = 2)	Horse (n = 5)	Human (n = 10)	Pig (n = 6)	Possum (n = 2)	Rabbit (n = 3)	Sheep (n = 2)	Waterfowl (n = 11)
**Observed species**	334 (180)	416 (129)	408 (96)	377 (55)	**1244** **(179)**	290 (77)	**1224** **(63)**	1023 (276)	**998** **(458)**	**861** **(88)**	532 (242)	571 (64)	**1201** **(70)**	375 (174)
**Shannon**	3.85 (1.05)	3.77 (1.38)	**5.41** **(0.67)**	5.09 (0.37)	**8.26** **(0.57)**	4.80 (0.82)	**8.21** **(0.11)**	6.36 (0.77)	**6.38** **(1.15)**	**7.25** **(0.19)**	**6.04** **(0.69)**	**6.40** **(0.28)**	**8.06** **(0.62)**	4.17 (6.98)
**Simpson**	0.83 (0.08)	0.76 (0.15)	0.93 (0.03)	0.93 (0.02)	**0.99** **(0.005)**	0.91 (0.04)	**0.99** **(0.0004)**	0.94 (0.04)	0.95 (0.03)	**0.98** **(0.002)**	0.96 (0.01)	**0.97** **(0.006)**	**0.94** **(0.003)**	0.85 (0.09)
**Evenness**^b^	0.006 (0.004)	0.004 (0.001)	0.003 (0.001)	0.003 (0.001)	**0.001** **(2x10**^**-4**^**)**	0.004 (0.001)	8x10^-4^ (5x10^-5^)	0.001 (3x10^-4^)	0.002 (0.002)	0.001 (1x10^-4^)	0.002 (0.001)	0.002 (2x10^-4^)	0.001 (5x10^-5^)	0.004 (0.002)

^a^Alpha diversity indices calculated at rarefaction depth of 20000 sequences. Standard deviation of each mean presented in parenthesis. Data in bold represents diversity indices that are significantly (P < 0.05) different from those observed for *Pteropus poliocephalus*.

^b^Evenness of each sample as calculated by Simpson Equitability index

**Fig 3:**
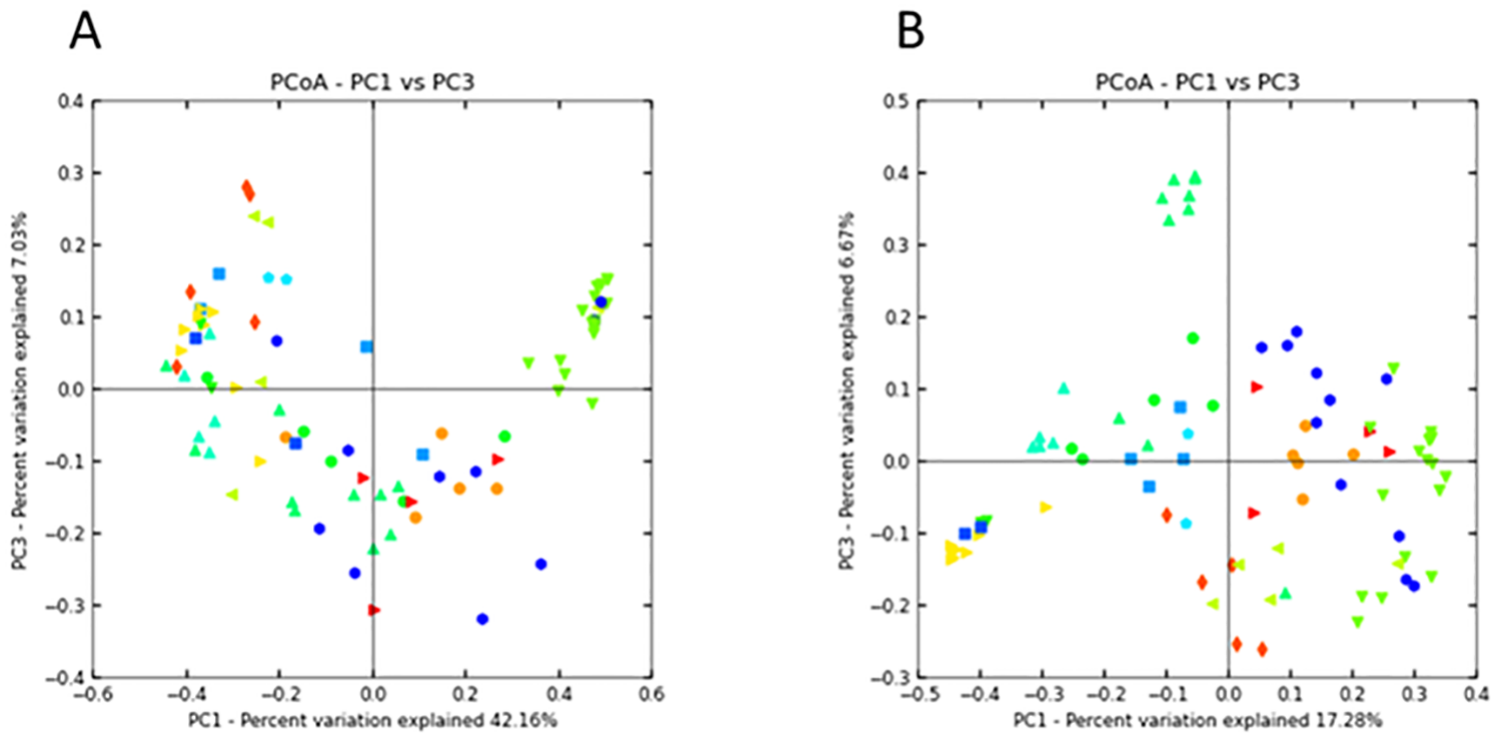
Principal co-ordinate analysis (PCoA) of microbiome faecal diversity of regional animal species. Plots were generated using A) weighted and B) unweighted UniFrac metrics. Analysis included *Pteropus poliocephalus* (n = 18; green triangle), humans (n = 10; aqua triangles), possum (n = 2; light blue dot), sheep (n = 2; dark blue square), horse (n = 5; green dot, rabbit (n = 3; blue square), dog (n = 5; lime triangle), birds (n = 4; red triangle), cows (n = 8; yellow triangle), pigs (n = 6; light blue triangle, cat (n = 5; red diamonds), chicken (n = 5; orange circle), goats (n = 2; green triangle) and waterfowl (n = 11; dark blue).

Principal co-ordinate analysis using weighted and unweighted UniFrac metrics was conducted to define relationships between the different faecal microbiomes (Fig 3). *P*. *poliocephalus* samples clustered together but also showed two location-dependent sub-clusters. Waterfowl faeces were observed to group closely with bat samples, especially those from Yarra Bend. This was particularly evident for the weighted UniFrac analysis, suggesting relationships in the relative abundance of the identified taxa. Human faecal microbiome samples clustered together and away from other animal groups in unweighted Unifrac plots. However, this separation was lost when the relative abundance of OTUs was accounted for using the weighted Unifrac metric.

**Fig 4:**
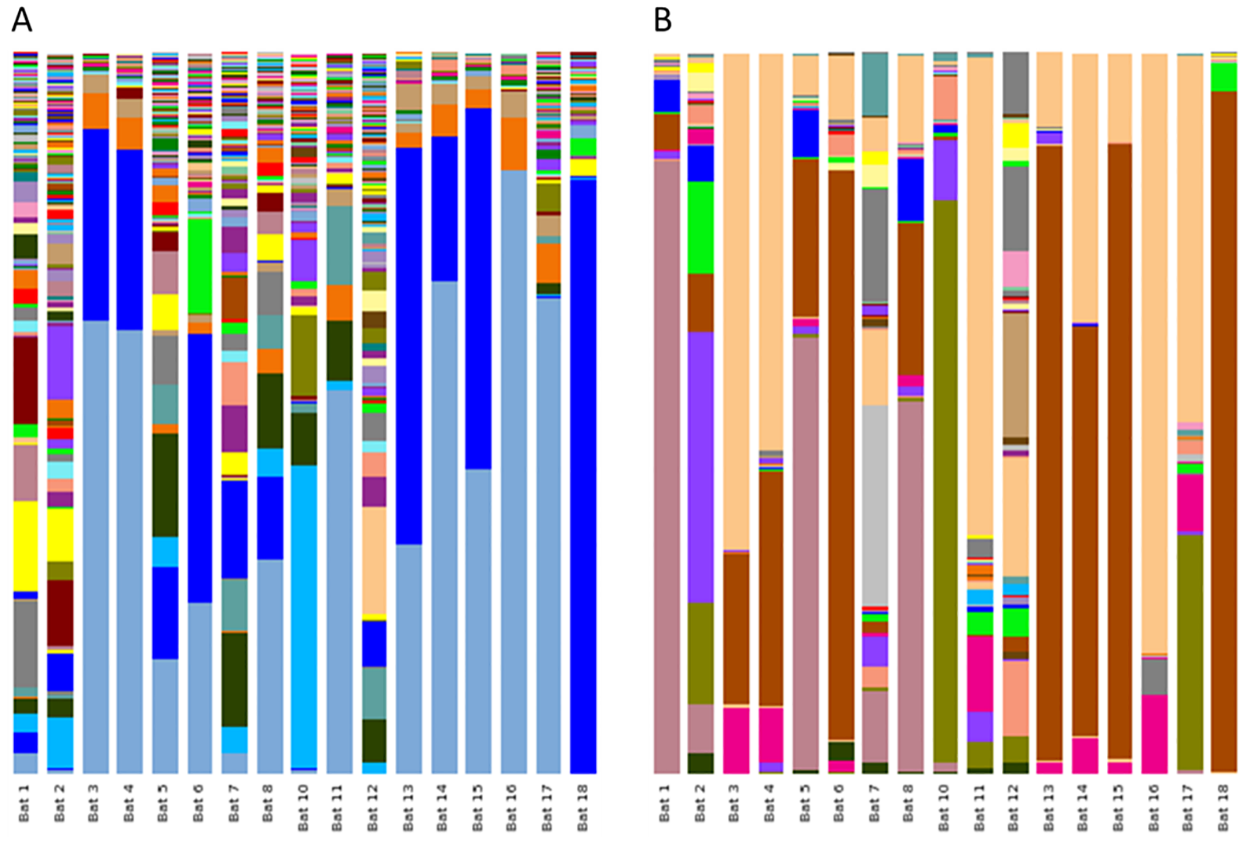
Species level bacterial community profile of faecal microbiome of *Pteropus poliocephalus*. A) Taxonomic classification based on Genbank database (access date 02/09/2016). B) Taxonomic classification based on GreenGenes 13_8 release. Bat faeces samples 1–13 collected from Yarra Bend (Victoria, Melbourne). Bat faeces samples 14–18 collected from Doveton (Victoria, Australia). Taxonomic legends and relative proportions are provided in S5 File.

*Identification of zoonotic bacterial pathogens within* Pteropus poliocephalus *populations*. To aid in species level identification, secondary taxonomic classification was undertaken using a Genbank 16S rRNA reference library. Closed reference OTU-picking resulted in the attribution of 1,237,456 sequences into 7824 taxa (68.1%; samples mean ± SD of 68747 ± 23367). In contrast to GreenGenes classification, only ~0.1% of sequences were identified as singletons and doubletons, leaving 1467 identified OTUs. Of these, 639 contained >10 sequences per OTU and were classified to the species level (S1 File); GreenGenes classification identified only 79 species under the same criteria. Significant differences were observed between the species level communities classified by Genbank and GreenGenes reference libraries (Fig 4). In general, classifications using the Genbank database predicted a higher level of species diversity within the samples.

**Fig 5:**
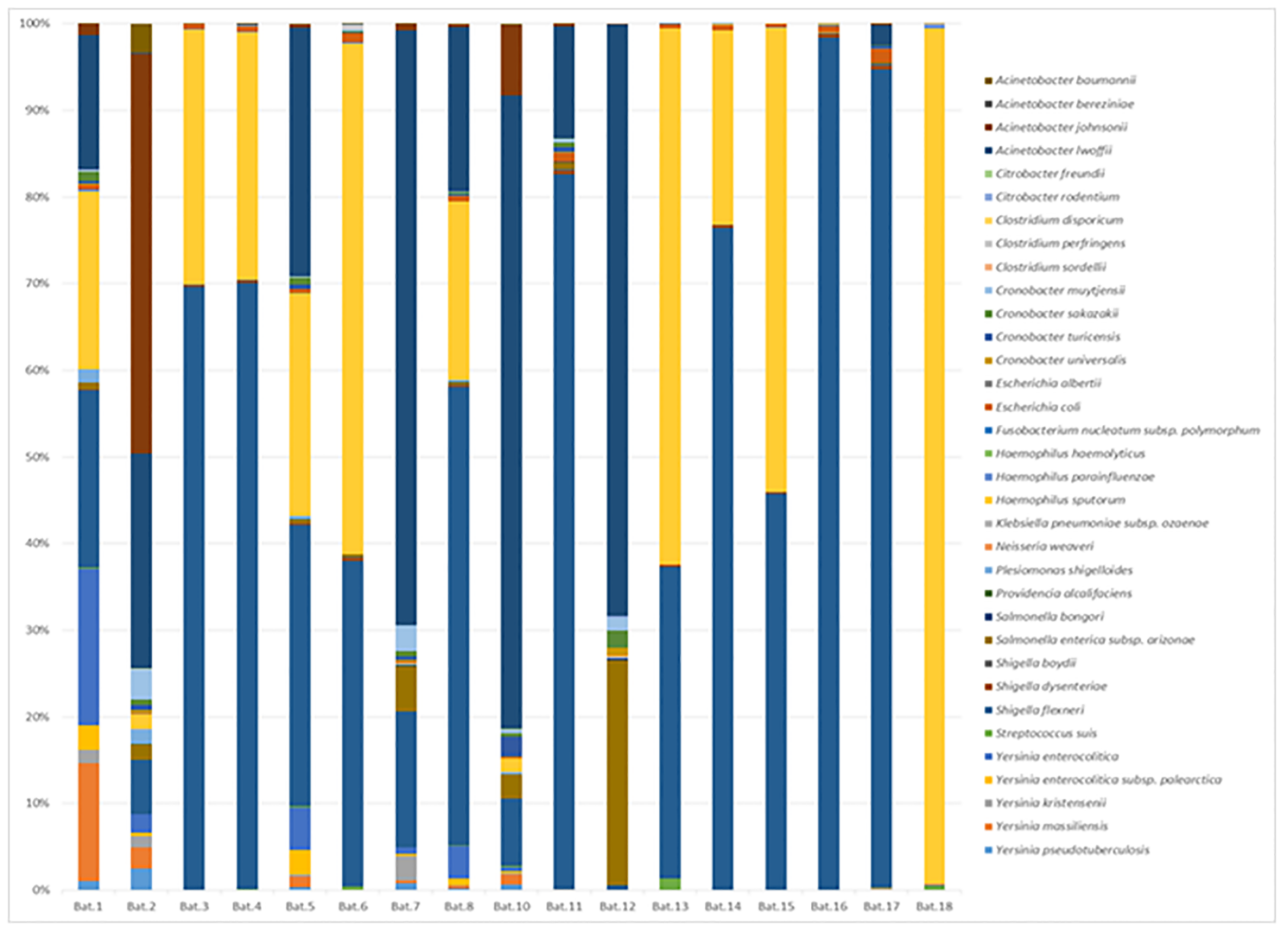
Potential human pathogens identified within *Pteropus poliocephalus* faeces. Data was obtained using QIIME 1.8.0 closed-reference OTU assignment using Genbank 16S rRNA database (accessed 02/09/2016). Confirmation of pathogen status was conducted using a dual literature survey approach.

Analysis of the 639 OTUs represented by >10 sequences within the *Pteropid* faeces, identified 33 potential food- or community-borne pathogens representing 15 genera (Fig 5 and S1 File). Alignment of raw sequence reads for each identified pathogen to the SILVA, RDP and GreenGenes databases was conducted to validate designated OTU classifications. Classification below family level could not be obtained for amplicons classified as *Providencia*, *Plesiomonas* and *Neisseria*. Thus, these genera were subsequently removed from further investigations.

*In silico* PIM analysis was conducted on representative species for each genus (Table 3 and S4 File). The threshold for differentiation of species within a genus was set at 97%. Thus matrix results of ≥97%, representing values within the QIIME clustering threshold, indicated taxa that could not be defined to the species level. Based on this threshold only *C*. *sordellii* could be unambiguously identified at a 97% threshold from all other clostridial species (Table 3). It is important to note however, that *C*. *perfringens* could be defined from all other species with the exception of *C*. *maximum* which was at the clustering threshold of 97%; suggesting differentiation of this pathogen may be possible. Further, supporting this was the results of Taxonomer (S4 File) which characterised 10 sequences as belonging to this species. The following pathogens could not be differentiated from other species within their genus: *Shigella* spp., *Escherichia* spp., *A*. *baumannii*, *A*. *bereziniae*, *A*. *johnsonii*, *C*. *disporicum*, *F*. *nucleatum*, *H*. *influenza*, *H*. *parainfluenzae*, *Citrobacter* spp., *Cronobacter* spp. or *Klebsiella* spp. (with the exception of *K*. *variicola*) (Table 3). For sequences matching *Haemophilus*, *Yersinia*, *Salmonella*, *Fusobacterium* and *Streptococcus*, a degree of species-level classification could be achieved which enabled a level of species clustering (Table 3; S4 File). For example, within *Haemophilus*, *H*. *parainfluenza* reference amplicons showed V3-4 rRNA sequence differences compared to *H*. *influenza-H*. *haemolyticus*, and therefore could be accurately separated from these two species. PIM analysis supported this result (S4 File). However, *H*. *parainfluenzae* shared >97% sequence identity with *H*. *pittmaniae* and *H*. *sputorum*, and thus each of these species could not be accurately separated.

**Table 3:** PIM analyses for potential human pathogens that were identified by Genbank and met secondary confirmation criteria.

Genera	# Species	Identified Pathogenic Species (IPS)	# other species that match this species with ≥ 97% similarity^a^
***Acinetobacter***	52	*A*. *baumannii* *A*. *bereziniae* *A*. *johnsonii* *A*. *lwoffii*	12 10 13 5
***Citrobacter***	12	*C*. *freundii* *C*. *rodentium*	11 11
***Clostridia***	57	*C*. *disporicum* *C*. *perfringens* *C*. *sordellii*	5 1 0
***Cronobacter***	7	*C*. *muytjensii* *C*. *sakazakii* *C*. *turicensis* *C*. *universalis*	3 3 6 2
***Escherichia***	6	*E*. *albertii* *E*. *coli*	3 3
***Fusobacterium***	15	*F*. *nucleatum*	9
***Haemphilus***	9	*H*. *haemolyticus* *H*. *influenzae* *H*. *parainfluenzae* *H*. *sputorum*	3 3 2 2
***Klebsiella***	6	*K*. *pneumoniae*	4
***Salmonella***	2	*S*. *bongori* *S*. *enterica*	1 1
***Shigella***	4	*S*. *boydii* *S*. *dysenteriae* *S*. *flexneri*	1 1 1
***Streptococcus***	77	*S*. *suis*	9
***Yersinia***	17	*Y*. *enterocolitica* *Y*. *kristensenii* *Y*. *pseudotuberculosis*	12 11 5

^a^The number of investigated species, from each genus, in which total sequence identity across the ~450bp V4-5 amplicon was greater than 96.99%; the lower clustering threshold for unambiguous species classification. The full species list and PIM analysis data is presented in S4 File.

For *Salmonella* spp. a different affect was observed. Raw sequence reads classified as *S*. *enterica* shared 100% identity with *S*. *enterica* and *S*. *bongori* reference amplicons. However, *S*. *bongori* classified sequences had a high level of heterogeneity and could not be classified based on alignment to the selected references. It was hypothesised that the application of the 97% classification threshold resulted in the clustering of divergent species under this OTU.

**Fig 6:**
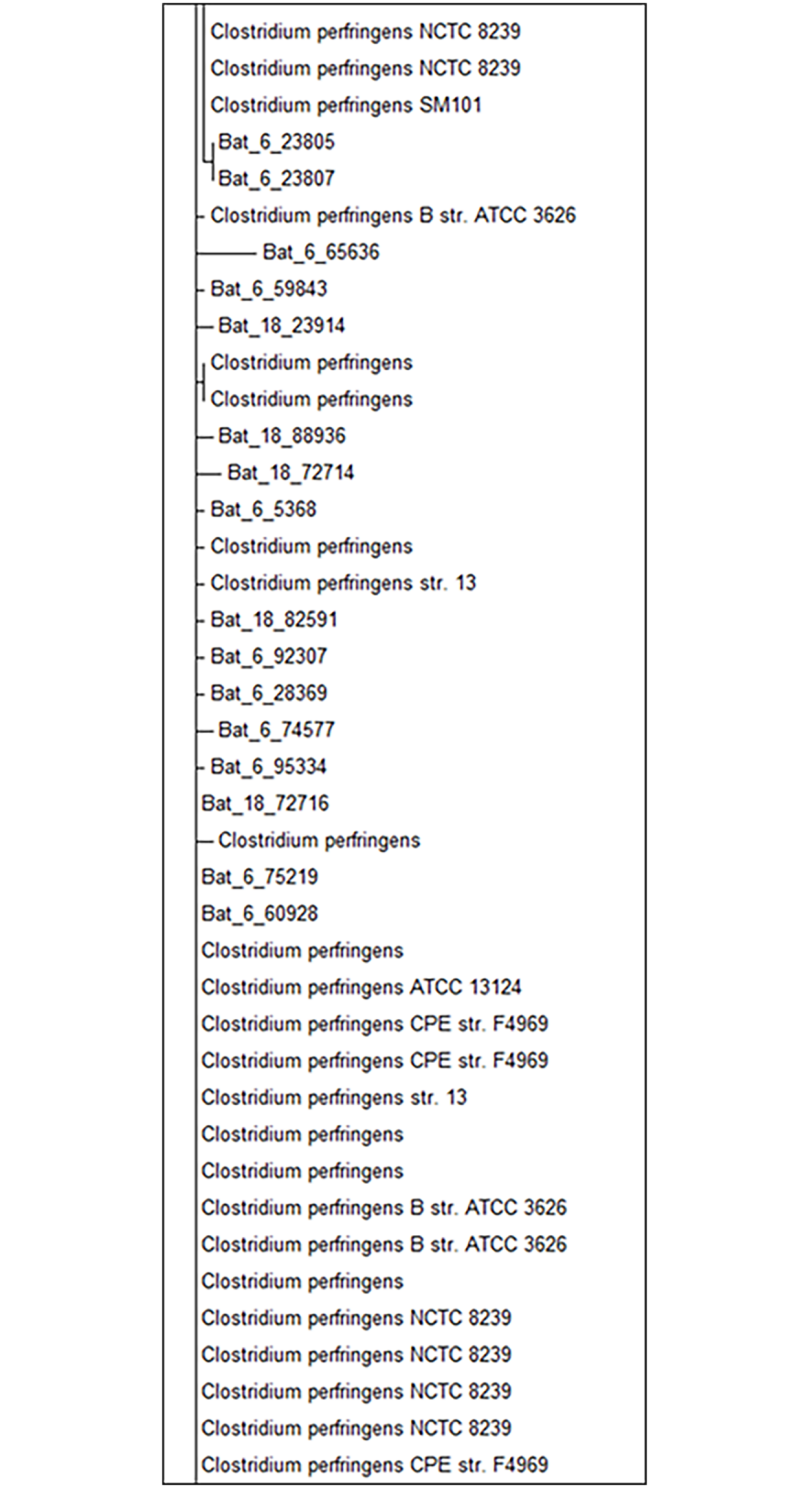
Maximum-likelihood tree of the relationship between V3-V4 amplicons derived from *Pteropus poliocephalus* to that of references sequences from *Clostridium perfringens*. Full tree presented in S3 File.

In total, 99% of sequences classified as *C*. *perfringens* shared significant identity to the associated reference amplicon (Fig 6; full tree presented in S3 File). Further, BLAST analysis on the raw sequences provided secondary confirmation of these results, with reads achieving expect values <E 1x10^-100^ with >85% identity to *C*. *perfringens* (S6 File). Five animals were found to carry these sequences. However, 97% of the sequence reads were derived from two bats (Bat-6 and Bat-18). As Bat-18 was the sample with the lowest moisture content, Spearman correlative analysis was conducted to determine if a significant relationship existed between moisture content and the detection of *C*. *perfringens* and/or *Clostridium* spp. However, no significant relationship was observed.

## Discussion

The current study analysed the faecal microbiome of *Pteropus poliocephalus* bats using 16S rRNA amplicon sequencing and analysis methods as recommended by Caporaso et al. (2010). Sequences were clustered at 97% and OTU assignment was based on GreenGenes and Genbank database assignment. A negative control to account for any sequences derived from the DNA extraction kits/solutions was not included with the bat faecal sample sequencing. However, these were included with sequencing of faeces derived from other animal species. Data from these, and previous studies, demonstrated that despite the presence of amplified sequences, the amplicons associated with the pathogens of interest were not present within the negative control. Further, as samples were not considered to have low biomass, DNA extraction-associated sequences were not removed during further analysis [41].

As the focus of this study was to investigate the flying fox faecal microbiome, with the aim of identifying bacterial pathogens, closed OTU picking strategies were employed. It is recognised that bats may have a significant, but as yet undefined, role in the transmission of novel bacterial pathogens which may have been overlooked in the current study [15]. However, investigation of the open OTU data would not have identified any further known pathogens. Instead, studies utilising whole genome metagenomics would be required; such studies represent a focus for future research.

Of the 18 investigated faecal deposits, one sample failed to produce sufficient data for inclusion in the study. Of the remaining faeces, 0.2% of total sequence reads generated were identified as singletons and doubletons. This accounted for 95.44% of the observed taxonomic diversity. It is important to note that the elimination of artefact amplicons by chimera removal was not conducted following clustering and OTU picking in this study [42, 43]. However, removal of sequences with ≤ 2 reads, followed by exclusion of OTUs represented by < 10 sequences, was deemed to be sufficient to limit the presence of these products within the dataset. Supporting this was the finding that, despite the application of two databases (Greengenes and GenBank) and associated differences in OTU picking strategies, a similar number of total OTUs were observed (1,473 and 1,467, respectively) within the datasets. Furthermore, phylogenetic analysis, through application of individual raw sequences, enabled individual assessment and identification of amplicons likely to be the result of spurious amplification errors. In this way, the application of a conservative, multi-tiered approach is recommended for species level identification of pathogens within a 16S rRNA amplicon dataset.

Three bacterial families were detected in high abundance across the bat colonies. Members of the *Enterobacteriacea* family have been previously recognised as major constituents of bat faeces [22, 44]. Further, previous studies have demonstrated a correlation between faecal moisture content and *Enterobacteriacea* concentration [45, 46]. This supports the results found in the present study, and suggests a role for species of this family as potential indicators of scat age.

*Moraxellaceae* (*Acinetobacter* spp.) and *Pasteurellaceae* (*Actinobacillus* spp.) families have not been previously identified within Pteroptid faeces. These organisms are recognised as common commensals of many avian and mammal species, and are generally found to be associated with the mucous membranes of the upper respiratory tracts [47–49]. As both bacterial families contain known zoonotic pathogens, the high proportion of these families in pteroptids could represent a direct human disease risk. However, further evaluation is required to identify the specific species present, and ensure that amplicons were derived from viable cells and not non-viable organisms passed from the respiratory tract and through the short digestive tract of this species.

Significant differences in α- and β-diversities were observed between the microbiomes of bats from the two different colonies. This finding was not unexpected, as many studies on mammalian faecal microbiome have demonstrated that alterations in diet, lifestyle and spatial distribution can alter microbial diversity [20, 21, 50, 51]. Further, a study by Kooser et al. [52] observed that external bat microflora also changed in relation to behaviour and roosting patterns. Though Pteroptids were not included within the Kooser et al. [52] study, it is hypothesised that their faecal microbiome may vary with the same conditions. The diversity differences were observed to be primarily driven by the presence/absence of specific OTUs. Inter-colony microbiome comparison identified organisms at the Yarra Bend colony for which amplified sequences were not obtained from the Doveton bats, such as *Salmonella* spp. and *Providencia* spp. However, it was observed that the 101 OTUs absent within the Doveton samples were inconsistently present at the Yarra colony, and were detected at proportions of ≤ 0.1%. Thus, further faecal sampling from Doveton would be required to determine if some of the identified differences were driven by the slightly smaller Doveton cohort size.

The microbiomes of the combined *P*. *poliocephalus* samples showed significantly different α-diversities and clustered separately from the human, sheep, cow and pig microbiomes on PCoA plots, consistent with previous observation [53]. Interestingly, flying fox and waterfowl faeces exhibited similar α- and β-diversities. The reasons for this remain unknown. However, both species, in particular Australian wood ducks, possess short intestinal tracts with very short feed-passage times, potentially accounting for some of the similarity the faecal communities in the microbiome [4, 54]. Furthermore, waterfowl and flying fox faecal samples were both collected from the Yarra River environment, suggesting the potential interchange of certain taxa due to geographical proximity. Both avian and flying fox populations investigated in this study displayed low to non-detectable levels of *Bacteroides* spp., an organism commonly applied as a microbial source tracking marker for faecal contamination. High concentrations of *Bacteroides* have been associated with gut health and diets rich in protein and animal fats [55]. As frugivores, flying foxes primarily consume fruit, pollen and nectar from native plant species, which may account for the absence of this genus within faecal microbiome. Similarly, waterfowl are largely herbivorous (though can consume small insects and anthropoids), which suggests that the absence of *Bacteriodes* may be directly related to the dearth of animal fats within the diets of these species. Irrespective of this, these results demonstrate the novel finding that, despite their evolutionary separation, a relationship exists between the gastrointestinal microbiome of some avian species and that of *P*. *poliocephalus*.

Species level classification through the use of the GreenGenes database at 97% clustering has been previously described [21, 22, 56, 57]. The current study also applied this methodology, as is recommended for use with the QIIME pipeline. However, there are a number of caveats that must be considered when applying 16S rRNA amplicon data under these conditions. These include:

1. Limitations in classification associated with the selected database (i.e. GreenGenes v13.5 contains sequences for 627 unique species while RDP and Genbank have >12,000 and >17,000 respectively) [33];
2. The application of 97% clustering for species identification. The basis for categorisation at this level was derived from studies which investigated taxonomic identity across the full-length of the 16S rRNA gene [58]. Thus, classification based on this clustering method may not be applicable to smaller regions within the same gene;
3. The application of small amplicons where inter-species sequence identity may be as high as 100% results in poor species resolution.

To counter some of these limitations, we used manual annotation and bioinformatics analysis of raw sequence reads, after application of single or multiple databases for primary classification, as has been recommended. [21, 56]. Other researchers have applied different approaches. For example, the application of technologies such as the Oxford Nanopore MinIon can produce long-read data, such that near full length 16S rRNA gene sequences can now be obtained. This negates the constraints of small amplicon data and increases phylogenetic resolution [59]. Uncertainty in the clustering method can be addressed by grouping OTUs using multiple levels of similarity (i.e. 97%, 99% and 100%) [43]. Some researchers have also advocated that sample extraction and sequencing should be independently duplicated, and that species level results only be considered when replicated within both datasets [57]. Future, larger studies should consider methods to limits these uncertainties through increasing clustering thresholds to above 97% using richer 16S database systems.

The data presented here provide an initial analysis of the potential public health risks associated with bacterial pathogens present in Grey headed flying fox faeces. However, limitations of the study included an inability to resolve most sequences to species level despite the use of multiple databases. However, a number of sequences could be confidently resolved to closely related pathogenic species clusters. The output from this work provides information key to directing future studies to characterise and quantify the concentration of bacteria; information essential to the application of quantitative microbial risk assessments (QMRAs) for public places affected by this species (i.e. urban parks, gardens, orchards and waterways).

Our multi-tiered analysis identified that the greatest species level differentiation could be achieved, using these methods, for *C*. *sordellii* and *C*. *perfringens*. For C. perfringens, the carriage rate of this pathogen within the investigated flying fox population was low, with 97% of the 19 sequences derived from two animals. It is important to note that the use of Taxonomer, further decreased this value to 10 sequences. This suggests possible low overall carriage rates among flying foxes. To further define the potential risk presented by *C*. *perfringens* within the Pteroptid faeces (for QMRA application), the infectious dose (ID) and potential mode of infection must also be considered. To date, the ID for this organism has not been defined. However, Johnson et al [60] reported that >10^8^ viable organisms within a mouthful of food will result in disease. This is significantly higher than the potential concentration present within the Pteropid faeces (accounting for weight and 16S rRNA copy number), with the oral route of transmission also highly unlikely in the context of public exposure in the outside environment. Furthermore, contact with *C*. *perfringens* from local soil environments is likely to present a higher risk [61]. Thus, in this context, the zoonotic disease risk of *C*. *perfringens* can be predicted as very low. In contrast, pathogenic species clusters, such as those observed for *Haemophilus* and *Salmonella*, may be more indicative of potential zoonotic disease risk.

*Haemophilus* spp. have 6 copies of the 16S rRNA gene [62]. Thus, when the average number of detected sequences per animal and weight per scat are accounted for, it can be hypothesised that up to ~9000 viable organisms may be present per gram of faeces. However, *P*. *poliocephalus* are rarely observed as solitary individuals, preferring instead to roost in large colonies of up to 30,000 individuals [63]. During the course of this study, faecal deposition loads from the colony were calculated (data not shown) suggesting that, during winter, up to 41 kg (wet weight) of faeces may be released daily by the Yarra Bend colony. In turn, this suggests that up to 4 x 10^8^ potentially viable *H*. *sputorum*-*H*.*parainfluenzae* could be released into the soil, sediments and abutting waterway each day. *Haemophilus* spp. are capable of transmission between individuals. However, alike *C*. *perfringens*, the ID and zoonotic transmissibility of these species have not been defined [64, 65]. Thus, further studies are required to fully quantify the zoonotic potential of the *Haemophilus* species present in *P*. *poiocephalus* faeces.

For *Salmonella*, it was calculated that each bat had the potential to carry ~108 viable *S*. *bongori*-*S*. *enterica*/gram (weight and 16S rRNA gene copy number adjusted). This in turn would result in a possible daily load of 4 x 10^6^ organisms. However, unlike *Haemophilus* spp., *Salmonella* spp. represent a much greater risk to public health, as extended survival in environmental settings, including estuaries such as the Yarra River, has been previously documented [66] [67, 68]. Thus, the risk of infection includes not only those who contact bat faeces directly, but also those who engage in recreational use of the affected waterway.

## Conclusion

*Pteropus poliocephalus* are recognised hosts for a range of zoonotic pathogens [15, 69, 70]. Within Australia, this species has primarily been associated with the transmission of viral pathogens such as Hendra and Lyssavirus. However, bacterial pathogen carriage, particularly within faecal deposits, has been relatively overlooked [70, 71].

Here we have employed 16S rRNA amplicon sequencing to gain an overview of the community composition of *P*. *poliocephalus* faeces to identify putative human bacterial zoonotic pathogens. Our analyses identified a specific bacterial pathogen, and together with proportional recovery and faecal loading estimates, allowed us to estimate the risk presented by pathogenic-species clusters. However, we suggest that studies applying this approach must be aware of its potential limitations; particularly when applied to QMRAs. We recommend taking a conservative approach to species-level classifications, and using multiple lines of evidence as we have done. This is essential to prevent over-estimation of the human-health risk presented by any animal or environment under investigation. Furthermore, studies to define the relationship between observed abundance (as defined by 16S rRNA amplicon sequencing) and culturable pathogen concentration, within the faeces, are required to assess the applicability of community-based sequencing approaches in future QMRA applications.

## Supporting information

S1 File Sample, OTU and pathogen lists. Tab 1 contains a description of faecal samples applied for this study. Tab 2 contains a list of bacterial OTUs characterised by Genbank analysis and determination of pathogenic potential and tab 3 is the list of shortlisted pathogens identified in this study.
(XLSX)

S2 File QIIME scripts. Document outlining the all scripts applied to QIIME to generate the results described within this manuscript.
(DOCX)

S3 File Maximum-likelihood phylogenetic trees. Maximum-Likelihood trees constructed based on the Tamura-Nei model generated by MEGA7.
(ZIPX)

S4 File PIM analysis. Independent Percent Identity Matrix (PIM) results for reference amplicons for each identified pathogenic genera.
(XLSX)

S5 File Taxonomic legends. Taxonomic legend for Fig 4.
(PDF)

S6 File Clostridia BLAST results. BLASTn analysis of C. perfringens raw sequence data.
(XLSX)
