# Supplementary material for: What’s the risk? Identifying potential human pathogens within grey-headed flying foxes faeces
Source: PLoS One. 2018 Jan 23;13(1):e0191301. doi: 10.1371/journal.pone.0191301 (PMC5779653; doi:10.1371/journal.pone.0191301)
Supplement: S2 File — Document outlining the all scripts applied to QIIME to generate the results described within this manuscript. (DOCX) [file pone.0191301.s002.docx]

# Supplementary Material 2: QIIME Scripts

**Associated Paper Details:**

Bacterial microbiota and human pathogens in the guts of Grey-headed flying foxes

Rebekah Henry^1^, Penelope Galbraith^1^, Scott Coutts^2^, Toby Prosser^3^, John Boyce^4^, David T. McCarthy^1#^.

1. Environmental and Public Health Microbiology Laboratory (EPHM Lab), Department of Civil Engineering, Monash University, Wellington Road, Clayton 3800, Victoria, Australia

2. Micromon, Dept. of Microbiology, Monash University, Wellington Road, Clayton 3800, Victoria, Australia

3. Melbourne Water, Latrobe St, Docklands 3008, Victoria, Australia

4. Department of Microbiology, Monash University, Wellington Road, Clayton 3800, Victoria, Australia.

^#^Corresponding author information:

David T. McCarthy: Environmental and Public Health Microbiology Laboratory, Department of Civil Engineering, Monash University, Wellington Road, Clayton 3800, Victoria, Australia; email: email: [david.mccarthy@monash.edu](mailto:david.mccarthy@monash.edu); Phone [+61 3 9905 5068](tel:%2B61%203%209905%205068); Fax [+61 3 9905 4944](tel:%2B61%203%209905%204944).

##### 03/10/2016

- BLAST Run with following conditions

parallel_pick_otus_blast.py -i /nfs/home/hpcmed/coutts/SHARED/micromon/metagenomics/bek/meta4_meta5_merged/meta4_meta5_merged.fna -o /nfs/home/hpcmed/coutts/SHARED/micromon/metagenomics/bek/meta4_meta5_merged/META4_5_MERGED_BLAST/blast_picked_otus -r /nfs/home/hpcmed/coutts/SHARED/micromon/metagenomics/greengenes/gg_13_8_otus/rep_set/97_otus.fasta -T --jobs_to_start 24 --blast_db /nfs/home/hpcmed/coutts/SHARED/micromon/metagenomics/bek/meta4_meta5_merged/db/sequence.fasta

- From blast table remove all otus that are present below 10 to remove possible sequencing errors.

filter_otus_from_otu_table.py -i /data/rebekah_data/bioms/run4_5/closed_BLAST/otu_table.biom -o /data/rebekah_data/bioms/run4_5/closed_BLAST/otu_table_n10.biom -n 10

biom convert -b -i /data/rebekah_data/bioms/run4_5/closed_BLAST/otu_table_n10.biom -o /data/rebekah_data/bioms/run4_5/closed_BLAST/otu_table_n10.txt

##### 04/10/2016

- Google drive folder has details of link between GI and species identifers
- First need to clean up the biom table to have only the gi numbers and no other details

sed -i 's/ref.*NR_.*//g' otu_table.txt

sed -i 's/gi//g' otu_table.txt

biom convert -b -i otu_table.biom -o otu_table_full.txt

- Then in excel the numbers present in out_table.txt were placed over the OTUIDs in the out_table_full.txt. This file was then saves as out_table_gi_IDs.txt

SourceTrackerOTU2taxon otu_table_gi_IDs_transpose.txt NCBI_species.txt otu_table_converted.txt

- The output was then transposed in excel and samples that were not relevant to analysis removed (only bat samples retained)
- All samples with <10 sequences were manually removed
- The file was then converted to a biom

biom convert -i otu_table_converted_bat_only_taxa.txt -o otu_table_converted_bat_only_taxa.biom --table-type="OTU table"

- Biom summary conducted

biom summarize-table -i otu_table_converted_bat_only_taxa.biom -o otu_table_converted_bat_only_taxa_summary.txt

- Sample I removed due to low reads, and the samples ordered. Taxa summary then run

plot_taxa_summary.py -i otu_table_converted_bat_only_taxa.txt -o /data/rebekah_data/bat_study/BLAST/taxa_summary -c bar

- The otu_table_converted_bat_only_taxa.txt was then converted to an excel sheet and the genus level pathogens identified (manually – as issue with filter_taxa_from_otu_table.py)

##### 10/10/2016

- Oligotyping set up and identification of files
  - Used the google drive file provided by Dieter to make a tab delimited taxonomy file to link the gi numbers to the bacteria identification.
    - Important the details need to have a unique identifier number so identifier gi|699005452|ref|NR_126321.1 was shortened to 126321.1
  - The OTU map also needed to be altered to provide unique identifiers (the same format as above) for each of the sequences this was done by using:
    - sed -i 's/gi.*ref*//g' meta4_meta5_merged_otus.txt
    - sed -i 's/|NR_//g' meta4_meta5_merged_otus_altered.txt
    - sed -i 's/\|//g' meta4_meta5_merged_otus_altered.txt
- Oligotyping was then tested on C. sodellii

q2oligo.py taxonomy_ncbi_NR.txt meta4_meta5_merged_otus_altered.txt '113140.1'

- This step seems not to be specifically pulling the line…..have tried instead (sed to pull out the specific line and then write to a new file):

sed -n '/113140.1/w /data/rebekah_data/bat_study/BLAST/oligotyping/c_sordelii/113140.txt' /data/rebekah_data/bioms/run4_5/closed_BLAST/original/meta4_meta5_merged_otus_altered.txt

- This has pulled out the specific line and now running:

filter_fasta.py -f /data/rebekah_data/bioms/run4_5/open/meta4_meta5_merged.fna -m /data/rebekah_data/bat_study/BLAST/oligotyping/c_sordelii/113140.txt -o /data/rebekah_data/bat_study/BLAST/oligotyping/c_sordelii/113140.fasta

- Then to pull from the fasta file only the sequences asscoaited with the bats.

sed -n '/bat\|Bat/{p;n;p;}' /data/rebekah_data/bat_study/BLAST/oligotyping/c_sordelii/113140.fasta > output.txt

- Then meta data was stripped for use with oligotyping pipeline

stripMeta.py output.txt output_stripped.txt

### 11/10/2016

- Continue fasta sequence removal for all interested species

sed -n '/028685.1/w /data/rebekah_data/bat_study/BLAST/oligotyping/rodentium/028685.fasta' /data/rebekah_data/bioms/run4_5/closed_BLAST/original/meta4_meta5_merged_otus_altered.txt

filter_fasta.py -f /data/rebekah_data/bioms/run4_5/open/meta4_meta5_merged.fna -m /data/rebekah_data/bat_study/BLAST/oligotyping/rodentium/028685.fasta -o /data/rebekah_data/bat_study/BLAST/oligotyping/rodentium/028685_seqs.fasta

sed -n '/bat\|Bat/{p;n;p;}' /data/rebekah_data/bat_study/BLAST/oligotyping/rodentium/028685_seqs.fasta > /data/rebekah_data/bat_study/BLAST/oligotyping/rodentium/028685_seqs_bats.fasta

sed -n '/104930.1/w /data/rebekah_data/bat_study/BLAST/oligotyping/haemolyticus/samples.txt' /data/rebekah_data/bioms/run4_5/closed_BLAST/original/meta4_meta5_merged_otus_altered.txt

filter_fasta.py -f /data/rebekah_data/bioms/run4_5/open/meta4_meta5_merged.fna -m /data/rebekah_data/bat_study/BLAST/oligotyping/haemolyticus/samples.txt -o /data/rebekah_data/bat_study/BLAST/oligotyping/haemolyticus/104930.fasta

sed -n '/bat\|Bat/{p;n;p;}' /data/rebekah_data/bat_study/BLAST/oligotyping/haemolyticus/104930.fasta > /data/rebekah_data/bat_study/BLAST/oligotyping/haemolyticus/104930_bats.fasta

sed -n '/026331.1/w /data/rebekah_data/bat_study/BLAST/oligotyping/shigella_escherichia/flexneri/samples026331.txt' /data/rebekah_data/bioms/run4_5/closed_BLAST/original/meta4_meta5_merged_otus_altered.txt

filter_fasta.py -f /data/rebekah_data/bioms/run4_5/open/meta4_meta5_merged.fna -m /data/rebekah_data/bat_study/BLAST/oligotyping/shigella_escherichia/flexneri/samples026331.txt -o /data/rebekah_data/bat_study/BLAST/oligotyping/shigella_escherichia/flexneri/026331.fasta

sed -n '/bat\|Bat/{p;n;p;}' /data/rebekah_data/bat_study/BLAST/oligotyping/shigella_escherichia/flexneri/026331.fasta > /data/rebekah_data/bat_study/BLAST/oligotyping/shigella_escherichia/flexneri/026331_bats.fasta

sed -n '/118143.1/w /data/rebekah_data/bat_study/BLAST/oligotyping/sputorum/118143.txt' /data/rebekah_data/bioms/run4_5/closed_BLAST/original/meta4_meta5_merged_otus_altered.txt

filter_fasta.py -f /data/rebekah_data/bioms/run4_5/open/meta4_meta5_merged.fna -m /data/rebekah_data/bat_study/BLAST/oligotyping/sputorum/118143.txt -o /data/rebekah_data/bat_study/BLAST/oligotyping/sputorum/118143.fasta

sed -n '/bat\|Bat/{p;n;p;}' /data/rebekah_data/bat_study/BLAST/oligotyping/sputorum/118143.fasta > /data/rebekah_data/bat_study/BLAST/oligotyping/sputorum/118143_bats.fasta

sed -n '/117625.1/w /data/rebekah_data/bat_study/BLAST/oligotyping/bereziniae/117625.txt' /data/rebekah_data/bioms/run4_5/closed_BLAST/original/meta4_meta5_merged_otus_altered.txt

filter_fasta.py -f /data/rebekah_data/bioms/run4_5/open/meta4_meta5_merged.fna -m /data/rebekah_data/bat_study/BLAST/oligotyping/bereziniae/117625.txt -o /data/rebekah_data/bat_study/BLAST/oligotyping/bereziniae/117625.fasta

sed -n '/bat\|Bat/{p;n;p;}' /data/rebekah_data/bat_study/BLAST/oligotyping/bereziniae/117625.fasta > /data/rebekah_data/bat_study/BLAST/oligotyping/bereziniae/117625_bats.fasta

sed -n '/028894.1/w /data/rebekah_data/bat_study/BLAST/oligotyping/freundii/028894.txt' /data/rebekah_data/bioms/run4_5/closed_BLAST/original/meta4_meta5_merged_otus_altered.txt

filter_fasta.py -f /data/rebekah_data/bioms/run4_5/open/meta4_meta5_merged.fna -m /data/rebekah_data/bat_study/BLAST/oligotyping/freundii/028894.txt -o /data/rebekah_data/bat_study/BLAST/oligotyping/freundii/028894.fasta

sed -n '/bat\|Bat/{p;n;p;}' /data/rebekah_data/bat_study/BLAST/oligotyping/freundii/028894.fasta > /data/rebekah_data/bat_study/BLAST/oligotyping/freundii/028894_bats.fasta

sed -n '/042053.1/w /data/rebekah_data/bat_study/BLAST/oligotyping/alcalifaciens/042053.txt' /data/rebekah_data/bioms/run4_5/closed_BLAST/original/meta4_meta5_merged_otus_altered.txt

filter_fasta.py -f /data/rebekah_data/bioms/run4_5/open/meta4_meta5_merged.fna -m /data/rebekah_data/bat_study/BLAST/oligotyping/alcalifaciens/042053.txt -o /data/rebekah_data/bat_study/BLAST/oligotyping/alcalifaciens/042053.fasta

sed -n '/bat\|Bat/{p;n;p;}' /data/rebekah_data/bat_study/BLAST/oligotyping/alcalifaciens/042053.fasta > /data/rebekah_data/bat_study/BLAST/oligotyping/alcalifaciens/042053_bats.fasta

sed -n '/026209.1/w /data/rebekah_data/bat_study/BLAST/oligotyping/lwoffi/026209.txt' /data/rebekah_data/bioms/run4_5/closed_BLAST/original/meta4_meta5_merged_otus_altered.txt

filter_fasta.py -f /data/rebekah_data/bioms/run4_5/open/meta4_meta5_merged.fna -m /data/rebekah_data/bat_study/BLAST/oligotyping/lwoffi/026209.txt -o /data/rebekah_data/bat_study/BLAST/oligotyping/lwoffi/026209.fasta

sed -n '/bat\|Bat/{p;n;p;}' /data/rebekah_data/bat_study/BLAST/oligotyping/lwoffi/026209.fasta > /data/rebekah_data/bat_study/BLAST/oligotyping/lwoffi/026209_bats.fasta

sed -n '/115943.1/w /data/rebekah_data/bat_study/BLAST/oligotyping/universalis/115943.txt' /data/rebekah_data/bioms/run4_5/closed_BLAST/original/meta4_meta5_merged_otus_altered.txt

filter_fasta.py -f /data/rebekah_data/bioms/run4_5/open/meta4_meta5_merged.fna -m /data/rebekah_data/bat_study/BLAST/oligotyping/universalis/115943.txt -o /data/rebekah_data/bat_study/BLAST/oligotyping/universalis/115943.fasta

sed -n '/bat\|Bat/{p;n;p;}' /data/rebekah_data/bat_study/BLAST/oligotyping/universalis/115943.fasta > /data/rebekah_data/bat_study/BLAST/oligotyping/universalis/115943_bats.fasta

sed -n '/025902.1/w /data/rebekah_data/bat_study/BLAST/oligotyping/weaveri/025902.txt' /data/rebekah_data/bioms/run4_5/closed_BLAST/original/meta4_meta5_merged_otus_altered.txt

filter_fasta.py -f /data/rebekah_data/bioms/run4_5/open/meta4_meta5_merged.fna -m /data/rebekah_data/bat_study/BLAST/oligotyping/weaveri/025902.txt -o /data/rebekah_data/bat_study/BLAST/oligotyping/weaveri/025902.fasta

sed -n '/bat\|Bat/{p;n;p;}' /data/rebekah_data/bat_study/BLAST/oligotyping/weaveri/025902.fasta > /data/rebekah_data/bat_study/BLAST/oligotyping/weaveri/025902_bats.fasta

sed -n '/026491.1/w /data/rebekah_data/bat_study/BLAST/oligotyping/disporicum/026491.txt' /data/rebekah_data/bioms/run4_5/closed_BLAST/original/meta4_meta5_merged_otus_altered.txt

filter_fasta.py -f /data/rebekah_data/bioms/run4_5/open/meta4_meta5_merged.fna -m /data/rebekah_data/bat_study/BLAST/oligotyping/disporicum/026491.txt -o /data/rebekah_data/bat_study/BLAST/oligotyping/disporicum/026491.fasta

sed -n '/bat\|Bat/{p;n;p;}' /data/rebekah_data/bat_study/BLAST/oligotyping/disporicum/026491.fasta > /data/rebekah_data/bat_study/BLAST/oligotyping/disporicum/026491_bats.fasta

sed -n '/112169.1/w /data/rebekah_data/bat_study/BLAST/oligotyping/perfringens/112169.txt' /data/rebekah_data/bioms/run4_5/closed_BLAST/original/meta4_meta5_merged_otus_altered.txt

filter_fasta.py -f /data/rebekah_data/bioms/run4_5/open/meta4_meta5_merged.fna -m /data/rebekah_data/bat_study/BLAST/oligotyping/perfringens/112169.txt -o /data/rebekah_data/bat_study/BLAST/oligotyping/perfringens/112169.fasta

sed -n '/bat\|Bat/{p;n;p;}' /data/rebekah_data/bat_study/BLAST/oligotyping/perfringens/112169.fasta > /data/rebekah_data/bat_study/BLAST/oligotyping/perfringens/112169_bats.fasta

sed -n '/025569.1/w /data/rebekah_data/bat_study/BLAST/oligotyping/shigella_escherichia/albertii/025569.txt' /data/rebekah_data/bioms/run4_5/closed_BLAST/original/meta4_meta5_merged_otus_altered.txt

filter_fasta.py -f /data/rebekah_data/bioms/run4_5/open/meta4_meta5_merged.fna -m /data/rebekah_data/bat_study/BLAST/oligotyping/shigella_escherichia/albertii/025569.txt -o /data/rebekah_data/bat_study/BLAST/oligotyping/shigella_escherichia/albertii/025569.fasta

sed -n '/bat\|Bat/{p;n;p;}' /data/rebekah_data/bat_study/BLAST/oligotyping/shigella_escherichia/albertii/025569.fasta > /data/rebekah_data/bat_study/BLAST/oligotyping/shigella_escherichia/albertii/025569_bats.fasta

sed -n '/044827.1/w /data/rebekah_data/bat_study/BLAST/oligotyping/shigellodies/044827.txt' /data/rebekah_data/bioms/run4_5/closed_BLAST/original/meta4_meta5_merged_otus_altered.txt

filter_fasta.py -f /data/rebekah_data/bioms/run4_5/open/meta4_meta5_merged.fna -m /data/rebekah_data/bat_study/BLAST/oligotyping/shigellodies/044827.txt -o /data/rebekah_data/bat_study/BLAST/oligotyping/shigellodies/044827.fasta

sed -n '/bat\|Bat/{p;n;p;}' /data/rebekah_data/bat_study/BLAST/oligotyping/shigellodies/044827.fasta > /data/rebekah_data/bat_study/BLAST/oligotyping/shigellodies/044827_bats.fasta

sed -n '/104901.1/w /data/rebekah_data/bat_study/BLAST/oligotyping/shigella_escherichia/boydii/104901.txt' /data/rebekah_data/bioms/run4_5/closed_BLAST/original/meta4_meta5_merged_otus_altered.txt

filter_fasta.py -f /data/rebekah_data/bioms/run4_5/open/meta4_meta5_merged.fna -m /data/rebekah_data/bat_study/BLAST/oligotyping/shigella_escherichia/boydii/104901.txt -o /data/rebekah_data/bat_study/BLAST/oligotyping/shigella_escherichia/boydii/104901.fasta

sed -n '/bat\|Bat/{p;n;p;}' /data/rebekah_data/bat_study/BLAST/oligotyping/shigella_escherichia/boydii/104901.fasta > /data/rebekah_data/bat_study/BLAST/oligotyping/shigella_escherichia/boydii/104901_bats.fasta

sed -n '/025158.1/w /data/rebekah_data/bat_study/BLAST/oligotyping/pseudotuberculosis/025158.txt' /data/rebekah_data/bioms/run4_5/closed_BLAST/original/meta4_meta5_merged_otus_altered.txt

filter_fasta.py -f /data/rebekah_data/bioms/run4_5/open/meta4_meta5_merged.fna -m /data/rebekah_data/bat_study/BLAST/oligotyping/pseudotuberculosis/025158.txt -o /data/rebekah_data/bat_study/BLAST/oligotyping/pseudotuberculosis/025158.fasta

sed -n '/bat\|Bat/{p;n;p;}' /data/rebekah_data/bat_study/BLAST/oligotyping/pseudotuberculosis/025158.fasta > /data/rebekah_data/bat_study/BLAST/oligotyping/pseudotuberculosis/025158_bats.fasta

sed -n '/025159.1/w /data/rebekah_data/bat_study/BLAST/oligotyping/kristensenii/025159.txt' /data/rebekah_data/bioms/run4_5/closed_BLAST/original/meta4_meta5_merged_otus_altered.txt

filter_fasta.py -f /data/rebekah_data/bioms/run4_5/open/meta4_meta5_merged.fna -m /data/rebekah_data/bat_study/BLAST/oligotyping/kristensenii/025159.txt -o /data/rebekah_data/bat_study/BLAST/oligotyping/kristensenii/025159.fasta

sed -n '/bat\|Bat/{p;n;p;}' /data/rebekah_data/bat_study/BLAST/oligotyping/kristensenii/025159.fasta > /data/rebekah_data/bat_study/BLAST/oligotyping/kristensenii/025159_bats.fasta

sed -n '/036918.1/w /data/rebekah_data/bat_study/BLAST/oligotyping/suis/036918.txt' /data/rebekah_data/bioms/run4_5/closed_BLAST/original/meta4_meta5_merged_otus_altered.txt

filter_fasta.py -f /data/rebekah_data/bioms/run4_5/open/meta4_meta5_merged.fna -m /data/rebekah_data/bat_study/BLAST/oligotyping/suis/036918.txt -o /data/rebekah_data/bat_study/BLAST/oligotyping/suis/036918.fasta

sed -n '/bat\|Bat/{p;n;p;}' /data/rebekah_data/bat_study/BLAST/oligotyping/suis/036918.fasta > /data/rebekah_data/bat_study/BLAST/oligotyping/suis/036918_bats.fasta

sed -n '/026332.1/w /data/rebekah_data/bat_study/BLAST/oligotyping/shigella_escherichia/dysenteriae/026332.txt' /data/rebekah_data/bioms/run4_5/closed_BLAST/original/meta4_meta5_merged_otus_altered.txt

filter_fasta.py -f /data/rebekah_data/bioms/run4_5/open/meta4_meta5_merged.fna -m /data/rebekah_data/bat_study/BLAST/oligotyping/shigella_escherichia/dysenteriae/026332.txt -o /data/rebekah_data/bat_study/BLAST/oligotyping/shigella_escherichia/dysenteriae/026332.fasta

sed -n '/bat\|Bat/{p;n;p;}' /data/rebekah_data/bat_study/BLAST/oligotyping/shigella_escherichia/dysenteriae/026332.fasta > /data/rebekah_data/bat_study/BLAST/oligotyping/shigella_escherichia/dysenteriae/026332_bats.fasta

sed -n '/104903.1/w /data/rebekah_data/bat_study/BLAST/oligotyping/palearctica/104903.txt' /data/rebekah_data/bioms/run4_5/closed_BLAST/original/meta4_meta5_merged_otus_altered.txt

filter_fasta.py -f /data/rebekah_data/bioms/run4_5/open/meta4_meta5_merged.fna -m /data/rebekah_data/bat_study/BLAST/oligotyping/palearctica/104903.txt -o /data/rebekah_data/bat_study/BLAST/oligotyping/palearctica/104903.fasta

sed -n '/bat\|Bat/{p;n;p;}' /data/rebekah_data/bat_study/BLAST/oligotyping/palearctica/104903.fasta > /data/rebekah_data/bat_study/BLAST/oligotyping/palearctica/104903_bats.fasta

sed -n '/044152.1/w /data/rebekah_data/bat_study/BLAST/oligotyping/massiliensis/044152.txt' /data/rebekah_data/bioms/run4_5/closed_BLAST/original/meta4_meta5_merged_otus_altered.txt

filter_fasta.py -f /data/rebekah_data/bioms/run4_5/open/meta4_meta5_merged.fna -m /data/rebekah_data/bat_study/BLAST/oligotyping/massiliensis/044152.txt -o /data/rebekah_data/bat_study/BLAST/oligotyping/massiliensis/044152.fasta

sed -n '/bat\|Bat/{p;n;p;}' /data/rebekah_data/bat_study/BLAST/oligotyping/massiliensis/044152.fasta > /data/rebekah_data/bat_study/BLAST/oligotyping/massiliensis/044152_bats.fasta

sed -n '/041750.1\|119276.1/w /data/rebekah_data/bat_study/BLAST/oligotyping/ozaenae/041750_119276.txt' /data/rebekah_data/bioms/run4_5/closed_BLAST/original/meta4_meta5_merged_otus_altered.txt

filter_fasta.py -f /data/rebekah_data/bioms/run4_5/open/meta4_meta5_merged.fna -m /data/rebekah_data/bat_study/BLAST/oligotyping/ozaenae/041750_119276.txt -o /data/rebekah_data/bat_study/BLAST/oligotyping/ozaenae/041750_119276.fasta

sed -n '/bat\|Bat/{p;n;p;}' /data/rebekah_data/bat_study/BLAST/oligotyping/ozaenae/041750_119276.fasta > /data/rebekah_data/bat_study/BLAST/oligotyping/ozaenae/041750_119276_bats.fasta

sed -n '/074888.1\|116124.1/w /data/rebekah_data/bat_study/BLAST/oligotyping/bongori/074888_116124.txt' /data/rebekah_data/bioms/run4_5/closed_BLAST/original/meta4_meta5_merged_otus_altered.txt

filter_fasta.py -f /data/rebekah_data/bioms/run4_5/open/meta4_meta5_merged.fna -m /data/rebekah_data/bat_study/BLAST/oligotyping/bongori/074888_116124.txt -o /data/rebekah_data/bat_study/BLAST/oligotyping/bongori/074888_116124.fasta

sed -n '/bat\|Bat/{p;n;p;}' /data/rebekah_data/bat_study/BLAST/oligotyping/bongori/074888_116124.fasta > /data/rebekah_data/bat_study/BLAST/oligotyping/bongori/074888_116124_bats.fasta

sed -n '/117842.1\|113141.1/w /data/rebekah_data/bat_study/BLAST/oligotyping/polymorphum/117842_113141.txt' /data/rebekah_data/bioms/run4_5/closed_BLAST/original/meta4_meta5_merged_otus_altered.txt

filter_fasta.py -f /data/rebekah_data/bioms/run4_5/open/meta4_meta5_merged.fna -m /data/rebekah_data/bat_study/BLAST/oligotyping/polymorphum/117842_113141.txt -o /data/rebekah_data/bat_study/BLAST/oligotyping/polymorphum/117842_113141.fasta

sed -n '/bat\|Bat/{p;n;p;}' /data/rebekah_data/bat_study/BLAST/oligotyping/polymorphum/117842_113141.fasta > /data/rebekah_data/bat_study/BLAST/oligotyping/polymorphum/117842_113141_bats.fasta

sed -n '/026206.1\|119358.1/w /data/rebekah_data/bat_study/BLAST/oligotyping/baumannii/026206_119358.txt' /data/rebekah_data/bioms/run4_5/closed_BLAST/original/meta4_meta5_merged_otus_altered.txt

filter_fasta.py -f /data/rebekah_data/bioms/run4_5/open/meta4_meta5_merged.fna -m /data/rebekah_data/bat_study/BLAST/oligotyping/baumannii/026206_119358.txt -o /data/rebekah_data/bat_study/BLAST/oligotyping/baumannii/026206_119358.fasta

sed -n '/bat\|Bat/{p;n;p;}' /data/rebekah_data/bat_study/BLAST/oligotyping/baumannii/026206_119358.fasta > /data/rebekah_data/bat_study/BLAST/oligotyping/baumannii/026206_119358_bats.fasta

sed -n '/042878.1\|118762.1/w /data/rebekah_data/bat_study/BLAST/oligotyping/parainfluenzae/042878_118762.txt' /data/rebekah_data/bioms/run4_5/closed_BLAST/original/meta4_meta5_merged_otus_altered.txt

filter_fasta.py -f /data/rebekah_data/bioms/run4_5/open/meta4_meta5_merged.fna -m /data/rebekah_data/bat_study/BLAST/oligotyping/parainfluenzae/042878_118762.txt -o /data/rebekah_data/bat_study/BLAST/oligotyping/parainfluenzae/042878_118762.fasta

sed -n '/bat\|Bat/{p;n;p;}' /data/rebekah_data/bat_study/BLAST/oligotyping/parainfluenzae/042878_118762.fasta > /data/rebekah_data/bat_study/BLAST/oligotyping/parainfluenzae/042878_118762_bats.fasta

sed -n '/102802.1\|044061.1/w /data/rebekah_data/bat_study/BLAST/oligotyping/turicensis/102802_044061.txt' /data/rebekah_data/bioms/run4_5/closed_BLAST/original/meta4_meta5_merged_otus_altered.txt

filter_fasta.py -f /data/rebekah_data/bioms/run4_5/open/meta4_meta5_merged.fna -m /data/rebekah_data/bat_study/BLAST/oligotyping/turicensis/102802_044061.txt -o /data/rebekah_data/bat_study/BLAST/oligotyping/turicensis/102802_044061.fasta

sed -n '/bat\|Bat/{p;n;p;}' /data/rebekah_data/bat_study/BLAST/oligotyping/turicensis/102802_044061.fasta > /data/rebekah_data/bat_study/BLAST/oligotyping/turicensis/102802_044061_bats.fasta

sed -n '/116125.1\|041696.1/w /data/rebekah_data/bat_study/BLAST/oligotyping/arizonae/116125_041696.txt' /data/rebekah_data/bioms/run4_5/closed_BLAST/original/meta4_meta5_merged_otus_altered.txt

filter_fasta.py -f /data/rebekah_data/bioms/run4_5/open/meta4_meta5_merged.fna -m /data/rebekah_data/bat_study/BLAST/oligotyping/arizonae/116125_041696.txt -o /data/rebekah_data/bat_study/BLAST/oligotyping/arizonae/116125_041696.fasta

sed -n '/bat\|Bat/{p;n;p;}' /data/rebekah_data/bat_study/BLAST/oligotyping/arizonae/116125_041696.fasta > /data/rebekah_data/bat_study/BLAST/oligotyping/arizonae/116125_041696_bats.fasta

sed -n '/114042.1\|024570.1/w /data/rebekah_data/bat_study/BLAST/oligotyping/shigella_escherichia/coli/114042_024570.txt' /data/rebekah_data/bioms/run4_5/closed_BLAST/original/meta4_meta5_merged_otus_altered.txt

filter_fasta.py -f /data/rebekah_data/bioms/run4_5/open/meta4_meta5_merged.fna -m /data/rebekah_data/bat_study/BLAST/oligotyping/shigella_escherichia/coli/114042_024570.txt -o /data/rebekah_data/bat_study/BLAST/oligotyping/shigella_escherichia/coli/114042_024570.fasta

sed -n '/bat\|Bat/{p;n;p;}' /data/rebekah_data/bat_study/BLAST/oligotyping/shigella_escherichia/coli/114042_024570.fasta > /data/rebekah_data/bat_study/BLAST/oligotyping/shigella_escherichia/coli/114042_024570_bats.fasta

sed -n '/041832.1\|104903.1/w /data/rebekah_data/bat_study/BLAST/oligotyping/enterocolitica/041832_104903.txt' /data/rebekah_data/bioms/run4_5/closed_BLAST/original/meta4_meta5_merged_otus_altered.txt

filter_fasta.py -f /data/rebekah_data/bioms/run4_5/open/meta4_meta5_merged.fna -m /data/rebekah_data/bat_study/BLAST/oligotyping/enterocolitica/041832_104903.txt -o /data/rebekah_data/bat_study/BLAST/oligotyping/enterocolitica/041832_104903.fasta

sed -n '/bat\|Bat/{p;n;p;}' /data/rebekah_data/bat_study/BLAST/oligotyping/enterocolitica/041832_104903.fasta > /data/rebekah_data/bat_study/BLAST/oligotyping/enterocolitica/041832_104903_bats.fasta

sed -n '/119114.1\|117624.1\|044975.1/w /data/rebekah_data/bat_study/BLAST/oligotyping/johnsonnii/119114_117624_044975.txt' /data/rebekah_data/bioms/run4_5/closed_BLAST/original/meta4_meta5_merged_otus_altered.txt

filter_fasta.py -f /data/rebekah_data/bioms/run4_5/open/meta4_meta5_merged.fna -m /data/rebekah_data/bat_study/BLAST/oligotyping/johnsonnii/119114_117624_044975.txt -o /data/rebekah_data/bat_study/BLAST/oligotyping/johnsonnii/119114_117624_044975.fasta

sed -n '/bat\|Bat/{p;n;p;}' /data/rebekah_data/bat_study/BLAST/oligotyping/johnsonnii/119114_117624_044975.fasta > /data/rebekah_data/bat_study/BLAST/oligotyping/johnsonnii/119114_117624_044975_bats.fasta

sed -n '/118088.1\|117437.1\|044059.1/w /data/rebekah_data/bat_study/BLAST/oligotyping/muytjensii/118088_117437_044059.txt' /data/rebekah_data/bioms/run4_5/closed_BLAST/original/meta4_meta5_merged_otus_altered.txt

filter_fasta.py -f /data/rebekah_data/bioms/run4_5/open/meta4_meta5_merged.fna -m /data/rebekah_data/bat_study/BLAST/oligotyping/muytjensii/118088_117437_044059.txt -o /data/rebekah_data/bat_study/BLAST/oligotyping/muytjensii/118088_117437_044059.fasta

sed -n '/bat\|Bat/{p;n;p;}' /data/rebekah_data/bat_study/BLAST/oligotyping/muytjensii/118088_117437_044059.fasta > /data/rebekah_data/bat_study/BLAST/oligotyping/muytjensii/118088_117437_044059_bats.fasta

sed -n '/118087.1\|118449.1\|113347.1\|044076.1/w /data/rebekah_data/bat_study/BLAST/oligotyping/sakazakii/118087_118449_113347_044076.txt' /data/rebekah_data/bioms/run4_5/closed_BLAST/original/meta4_meta5_merged_otus_altered.txt

filter_fasta.py -f /data/rebekah_data/bioms/run4_5/open/meta4_meta5_merged.fna -m /data/rebekah_data/bat_study/BLAST/oligotyping/sakazakii/118087_118449_113347_044076.txt -o /data/rebekah_data/bat_study/BLAST/oligotyping/sakazakii/118087_118449_113347_044076.fasta

- Strip meta data from fasta files for next stage of analysis

stripMeta.py /data/rebekah_data/bat_study/BLAST/oligotyping/bereziniae/117625_bats.fasta /data/rebekah_data/bat_study/BLAST/oligotyping/bereziniae/117625_bats_stripped.fasta

stripMeta.py /data/rebekah_data/bat_study/BLAST/oligotyping/alcalifaciens/042053_bats.fasta /data/rebekah_data/bat_study/BLAST/oligotyping/alcalifaciens/042053_bats_stripped.fasta

stripMeta.py /data/rebekah_data/bat_study/BLAST/oligotyping/arizonae/116125_041696_bats.fasta /data/rebekah_data/bat_study/BLAST/oligotyping/arizonae/116125_041696_bats_stripped.fasta

stripMeta.py /data/rebekah_data/bat_study/BLAST/oligotyping/baumannii/026206_119358_bats.fasta /data/rebekah_data/bat_study/BLAST/oligotyping/baumannii/026206_119358_bats_stripped.fasta

stripMeta.py /data/rebekah_data/bat_study/BLAST/oligotyping/bongori/074888_116124_bats.fasta /data/rebekah_data/bat_study/BLAST/oligotyping/bongori/074888_116124_bats_stripped.fasta

stripMeta.py /data/rebekah_data/bat_study/BLAST/oligotyping/disporicum/026491_bats.fasta /data/rebekah_data/bat_study/BLAST/oligotyping/disporicum/026491_bats_stripped.fasta

stripMeta.py /data/rebekah_data/bat_study/BLAST/oligotyping/enterocolitica/041832_104903_bats.fasta /data/rebekah_data/bat_study/BLAST/oligotyping/enterocolitica/041832_104903_bats_stripped.fasta

stripMeta.py /data/rebekah_data/bat_study/BLAST/oligotyping/freundii/028894_bats.fasta /data/rebekah_data/bat_study/BLAST/oligotyping/freundii/028894_bats_stripped.fasta

stripMeta.py /data/rebekah_data/bat_study/BLAST/oligotyping/haemolyticus/104930_bats.fasta /data/rebekah_data/bat_study/BLAST/oligotyping/haemolyticus/104930_bats_stripped.fasta

stripMeta.py /data/rebekah_data/bat_study/BLAST/oligotyping/johnsonnii/119114_117624_044975_bats.fasta /data/rebekah_data/bat_study/BLAST/oligotyping/johnsonnii/119114_117624_044975_bats_stripped.fasta

stripMeta.py /data/rebekah_data/bat_study/BLAST/oligotyping/kristensenii/025159_bats.fasta /data/rebekah_data/bat_study/BLAST/oligotyping/kristensenii/025159_bats_stripped.fasta

stripMeta.py /data/rebekah_data/bat_study/BLAST/oligotyping/lwoffi/026209_bats.fasta /data/rebekah_data/bat_study/BLAST/oligotyping/lwoffi/026209_bats_stripped.fasta

stripMeta.py /data/rebekah_data/bat_study/BLAST/oligotyping/massiliensis/044152_bats.fasta /data/rebekah_data/bat_study/BLAST/oligotyping/massiliensis/044152_bats_stripped.fasta

stripMeta.py /data/rebekah_data/bat_study/BLAST/oligotyping/muytjensii/118088_117437_044059_bats.fasta /data/rebekah_data/bat_study/BLAST/oligotyping/muytjensii/118088_117437_044059_bats_stripped.fasta

stripMeta.py /data/rebekah_data/bat_study/BLAST/oligotyping/ozaenae/041750_119276_bats.fasta /data/rebekah_data/bat_study/BLAST/oligotyping/ozaenae/041750_119276_bats_stripped.fasta

stripMeta.py /data/rebekah_data/bat_study/BLAST/oligotyping/palearctica/104903_bats.fasta /data/rebekah_data/bat_study/BLAST/oligotyping/palearctica/104903_bats_stripped.fasta

stripMeta.py /data/rebekah_data/bat_study/BLAST/oligotyping/parainfluenzae/042878_118762_bats.fasta /data/rebekah_data/bat_study/BLAST/oligotyping/parainfluenzae/042878_118762_bats_stripped.fasta

stripMeta.py /data/rebekah_data/bat_study/BLAST/oligotyping/perfringens/112169_bats.fasta /data/rebekah_data/bat_study/BLAST/oligotyping/perfringens/112169_bats_stripped.fasta

stripMeta.py /data/rebekah_data/bat_study/BLAST/oligotyping/polymorphum/117842_113141_bats.fasta /data/rebekah_data/bat_study/BLAST/oligotyping/polymorphum/117842_113141_bats_stripped.fasta

stripMeta.py /data/rebekah_data/bat_study/BLAST/oligotyping/pseudotuberculosis/025158_bats.fasta /data/rebekah_data/bat_study/BLAST/oligotyping/pseudotuberculosis/025158_bats_stripped.fasta

stripMeta.py /data/rebekah_data/bat_study/BLAST/oligotyping/rodentium/028685_seqs_bats.fasta /data/rebekah_data/bat_study/BLAST/oligotyping/rodentium/028685_seqs_bats_stripped.fasta

stripMeta.py /data/rebekah_data/bat_study/BLAST/oligotyping/sakazakii/118087_118449_113347_044076_bats.fasta /data/rebekah_data/bat_study/BLAST/oligotyping/sakazakii/118087_118449_113347_044076_bats_stripped.fasta

stripMeta.py /data/rebekah_data/bat_study/BLAST/oligotyping/shigella_escherichia/albertii/025569_bats.fasta /data/rebekah_data/bat_study/BLAST/oligotyping/shigella_escherichia/albertii/025569_bats_stripped.fasta

stripMeta.py /data/rebekah_data/bat_study/BLAST/oligotyping/shigella_escherichia/boydii/104901_bats.fasta /data/rebekah_data/bat_study/BLAST/oligotyping/shigella_escherichia/boydii/104901_bats_stripped.fasta

stripMeta.py /data/rebekah_data/bat_study/BLAST/oligotyping/shigella_escherichia/coli/114042_024570_bats.fasta /data/rebekah_data/bat_study/BLAST/oligotyping/shigella_escherichia/coli/114042_024570_bats_stripped.fasta

stripMeta.py /data/rebekah_data/bat_study/BLAST/oligotyping/shigella_escherichia/flexneri/026331_bats.fasta /data/rebekah_data/bat_study/BLAST/oligotyping/shigella_escherichia/flexneri/026331_bats_stripped.fasta

stripMeta.py /data/rebekah_data/bat_study/BLAST/oligotyping/shigellodies/044827_bats.fasta /data/rebekah_data/bat_study/BLAST/oligotyping/shigellodies/044827_bats_stripped.fasta

stripMeta.py /data/rebekah_data/bat_study/BLAST/oligotyping/sputorum/118143_bats.fasta /data/rebekah_data/bat_study/BLAST/oligotyping/sputorum/118143_bats_stripped.fasta

stripMeta.py /data/rebekah_data/bat_study/BLAST/oligotyping/suis/036918_bats.fasta /data/rebekah_data/bat_study/BLAST/oligotyping/suis/036918_bats_stripped.fasta

stripMeta.py /data/rebekah_data/bat_study/BLAST/oligotyping/turicensis/102802_044061_bats.fasta /data/rebekah_data/bat_study/BLAST/oligotyping/turicensis/102802_044061_bats_stripped.fasta

stripMeta.py /data/rebekah_data/bat_study/BLAST/oligotyping/universalis/115943_bats.fasta /data/rebekah_data/bat_study/BLAST/oligotyping/universalis/115943_bats_stripped.fasta

stripMeta.py /data/rebekah_data/bat_study/BLAST/oligotyping/weaveri/025902_bats.fasta /data/rebekah_data/bat_study/BLAST/oligotyping/weaveri/025902_bats_stripped.fasta

- Have downloaded and installed vsearch (<https://github.com/torognes/vsearch>) which will (among other things) identify sequences which are repeated within the fasta dataset – allowing us to highlight what could be real differences and match these against the blast database. Also to make better alignment trees and clustering.
- The common command for this tool is:

vsearch \

--derep_fulllength /data/rebekah_data/bat_study/BLAST/oligotyping/shigella_escherichia/flexneri/026331_bats_stripped_linear.fasta\

--sizeout \

--relabel_sha1 \

--fasta_width 0 \

--output /data/rebekah_data/bat_study/BLAST/oligotyping/shigella_escherichia/flexneri/026331_bats_stripped_linear_dereplicated.fasta

- This tool run on all fasta files to generate the figures on the number of sequence clusters

### 12/10/2016

- Using Silva did a primer probe test using our primers and then downloaded the sequences for all matching bacteria within the identified pathogen genera.
- Once this was completed then the sequences needed to be linearized prior to use and excess meta data stripped

awk 'NR==1 {print ; next} {printf /^>/ ? "\n"$0"\n" : $1} END {printf "\n"}' /data/rebekah_data/bat_study/Reference_sequences/acinetobacter.fasta > /data/rebekah_data/bat_study/Reference_sequences/acinetobacter_linear.fasta

stripMeta.py /data/rebekah_data/bat_study/Reference_sequences/acinetobacter_linear.fasta /data/rebekah_data/bat_study/Reference_sequences/acinetobacters_linear_stripped.fasta

- An Insilco PCR using Mothur then needs to be conducted to produce an output that is just from the region we are interested in within these sequences

pcr.seqs(fasta=/data/rebekah_data/bat_study/Reference_sequences/acinetobacters_linear_stripped.fasta, oligos=/data/rebekah_data/bat_study/Reference_sequences/primers.oligos)

- Once this was completed then the PCR products and the fasta for each bat were combined and clustalW run on unix in fast mode to enable comparison

awk 'NR==1 {print ; next} {printf /^>/ ? "\n"$0"\n" : $1} END {printf "\n"}' /data/rebekah_data/bat_study/Reference_sequences/fusobacterium.fasta > /data/rebekah_data/bat_study/Reference_sequences/fusobacterium_linear.fasta

stripMeta.py /data/rebekah_data/bat_study/Reference_sequences/fusobacterium_linear.fasta /data/rebekah_data/bat_study/Reference_sequences/fusobacterium_linear_stripped.fasta

sed -i 's/U/T/g' /data/rebekah_data/bat_study/Reference_sequences/fusobacterium/fusobacterium_linear_stripped.fasta

pcr.seqs(fasta=/data/rebekah_data/bat_study/Reference_sequences/fusobacterium/fusobacterium_linear_stripped.fasta, oligos=/data/rebekah_data/bat_study/Reference_sequences/primers.oligos)

- Strangely PCR products for the Mothur results were too large; not sure what this has occurred.
- Alternative in silco PCR tool applied – IPCRESS

ipcress -i /data/rebekah_data/bat_study/Reference_sequences/ipcress.txt -s /data/rebekah_data/bat_study/Reference_sequences/fusobacterium/fusobacterium_linear_stripped.fasta -m 3 -P > /data/rebekah_data/bat_study/Reference_sequences/fusobacterium/ipcress_pcr.txt

13/10/16

- To start to streamline these processes I have started to create and run shell scripts.
  - Once script is made need to activate by typing chmod +x file.sh

Script 1: ./U_TO_T_linear.sh (found in /data/rebekah_data/bat_study/Reference_sequences)

- - This script takes the reference sequence outputs from Silva, makes them linear and exchanges the U from the rRNA with a T; in preparation for use with IPCRESS.

Script 2: Then need to run through IPCRESS the PCR ./Ipcress.sh (found in /data/rebekah_data/bat_study/Reference_sequences)

- - This script runs Ipcress on the prepared files and then outputs this into a new folder

Script 3: Ipcress output needs to be cleaned to have only the sequence for alignment ipcress_fasta.sh (found in: /data/rebekah_data/bat_study/Reference_sequences/IPCRESS_PCR)

- - This script uses a series of sed commands to remove extra information from the file.

Script 4: Fasta files then run through clustalw using the clustalw.sh (found in /data/rebekah_data/bat_study/Reference_sequences/IPCRESS_PCR).

- - This script runs clustalw under the following conditions for all the input fasta sequences: clustalw -infile=$InFile1 -type=DNA -align -pwdnamatrix=clustalw -outorder=aligned -outfile=/data/rebekah_data/bat_study/Reference_sequences/ClustalW/results_out.aln -outputtree=nj -seed=10 -clustering=UPGMA –quiet

Script 5: Then to remove replicate sequences use vsearch.sh (found in /data/rebekah_data/bat_study/Reference_sequences/IPCRESS_PCR)

- - This script finds and identifies duplicate sequences so as to enable easier alignment of sequences.

Script 6: Then run Muscle alignment of sequences using muscel.sh (found in /data/rebekah_data/bat_study/Reference_sequences/Dereplicated).

- - This tool runs multiple alignments at once using muscle default parameters
  - I ran this tool on just the references sequences only as an initial guide
- I have then combined the references sequences (dereplicated; /data/rebekah_data/bat_study/Reference_sequences/Dereplicated) and the bat sequences for each potential pathogen (/data/rebekah_data/bat_study/BLAST/oligotyping) into a single fasta file (/data/rebekah_data/bat_study/BLAST/combined_ref_bats)
- Muscle.sh (altered to direct to specific folder) was then run, to allow an alignment of all sequences and to determine where the bat sequences sit among the reference sequences.
  - Due to size – this was run with nohup (only one core per analysis).

### 17/10/2016

- Have made new bash script to remove unknown bacteria from the reference sequences, so that trees and alignments are more meaningful for analysis

sed –e ‘/uncultured\|unknown\| sp./{N;d;}’ input > /data/rebekah_data/bat_study/Reference_sequences/reference_filtered/output.fasta

- Files then needed to go in an do further “clean-up” as there were many that had further un-related sequences present
- The titles were then fixed to shorten to genus and species level

sed -i 's/^.*;Streptococcus />Streptococcus /' /data/rebekah_data/bat_study/Reference_sequences/reference_filtered/streptococcus.fasta

- A second script was then run to change spaces between genus and species to underscores
  - This is important for the next IPCRESS PCR
- Remaining reference sequences then underwent IPCRESS PCR using the bash script as per above
  - Output in: /data/rebekah_data/bat_study/Reference_sequences/IPCRESS_PCR
- Ipcress output then converted to fasta using the previous script
  - Output in: /data/rebekah_data/bat_study/Reference_sequences/IPCRESS_PCR
    - This script has an added step now added to enable the titles to be retained
- Ipcress output then changed to make it linear using bash script
  - The output still in folder: /data/rebekah_data/bat_study/Reference_sequences/IPCRESS_PCR
- The bat sequences and the reference sequences were then combined into a single file and the folder stored at /data/rebekah_data/bat_study/BLAST/combined_ref_bats/filtered.
  - The only change to this was for shigella flexneri where, due to the number of sequences within the bat samples, representative sequences from each type within the bats were applied for sequence analysis.
- Muscle was conducted under default conditions (16 iterations) to enable alignment of the bat sequences to the reference sequences within the files. This was conducted using muscle.sh under nohup; due to the size and time for running
  - The output for this analysis is: /data/rebekah_data/bat_study/BLAST/combined_ref_bats/filtered/muscle_output

### 24/10/16

- Determining sequence number and presence of singletons/doubletons

split_otu_table.py -i /data/rebekah_data/bioms/run4_5/closed/otu_table.biom -o /data/rebekah_data/bioms/run4_5/closed/split/ -m /data/rebekah_data/bat_study/mapping/mapping4_5_Yarra_Doveton_bat.txt -f Project

biom summarize-table -i /data/rebekah_data/bioms/run4_5/closed/split/otu_table_Penny.biom -o /data/rebekah_data/bioms/run4_5/closed/split/otu_table_Penny_summary.txt

filter_otus_from_otu_table.py -i /data/rebekah_data/bioms/run4_5/closed/split/otu_table_Penny.biom -o /data/rebekah_data/bioms/run4_5/closed/split/otu_table_Penny_singleton_filtered.biom -n 2

biom summarize-table -i /data/rebekah_data/bioms/run4_5/closed/split/otu_table_Penny_singleton_filtered.biom -o /data/rebekah_data/bioms/run4_5/closed/split/otu_table_Penny_singleton_filtered_summary.txt

filter_otus_from_otu_table.py -i /data/rebekah_data/bioms/run4_5/closed/split/otu_table_Penny.biom -o /data/rebekah_data/bioms/run4_5/closed/split/otu_table_Penny_singleton_filtered.biom -n 3

biom summarize-table -i /data/rebekah_data/bioms/run4_5/closed/split/otu_table_Penny_singleton_filtered.biom -o /data/rebekah_data/bioms/run4_5/closed/split/otu_table_Penny_doubleton_filtered.txt

- Core diversity analysis re-run to omit singletons and doubletons from further analysis

core_diversity_analyses.py -i /data/rebekah_data/bioms/run4_5/closed/split/otu_table_Penny_doubleton_filtered.biom -p /data/rebekah_data/bioms/run4_5/closed/split/metrics.txt -m /data/rebekah_data/bioms/run4_5/closed/split/mapping_Penny.txt -e 40000 -t /data/rebekah_data/Yarra_Data/bioms/closed/pre-merged/97_otus.tree -o /data/rebekah_data/bat_study/core_diversity/

- Taxa summary edited and new taxa figure generated

plot_taxa_summary.py -i /data/rebekah_data/bat_study/core_diversity/taxa_plots/table_mc40000_sorted_L6.txt -o /data/rebekah_data/bat_study/core_diversity/taxa_plots/taxa_summary_plots/L6_edit

- Identify and plot most abundant taxa

filter_otu_from_otu_table.py -i /data/rebekah_data/bioms/run4_5/closed/split/otu_table_Penny_doubleton_filtered.biom -o /data/rebekah_data/bioms/run4_5/closed/split/otu_table_Penny_doubleton_filtered_05percent.biom --min_count_fraction 0.005

plot_taxa_summary.py -i /data/rebekah_data/bat_study/taxa_summaries/05_percent_bat_only/otu_table_Penny_doubleton_filtered_05percent_L6.txt -o /data/rebekah_data/bat_study/taxa_summaries/05_percent_bat_only/taxa_plot/ -c bar

### 25/10/16

- Need to repeat alpha diversity metric analysis on the closed OTU data (for Table 2 data)
  - This was repeated for each metric file

compare_alpha_diversity.py -i /data/rebekah_data/bat_study/core_diversity/arare_max40000/alpha_div_collated/simpson_e.txt -o /data/rebekah_data/bat_study/core_diversity/arare_max40000/alpha_div_collated/simpsone_compare.txt -m /data/rebekah_data/bioms/run4_5/closed/split/mapping_Penny.txt -c Sample_Location -t nonparametric -p fdr -n 1000

- Core microbioms computed to determine if difference between colonies.

compute_core_microbiome.py -i /data/rebekah_data/bioms/run4_5/closed/split/otu_table_Penny_doubleton_filtered.biom -o /data/rebekah_data/bat_study/core_microbiomes/re_run_all_bats

ompute_core_microbiome.py -i /data/rebekah_data/bioms/run4_5/closed/split/otu_table_Penny_doubleton_filtered.biom -o /data/rebekah_data/bat_study/core_microbiomes/re_run_colonies/ --mapping_fp /data/rebekah_data/bioms/run4_5/closed/split/mapping_Penny.txt --valid_states "Sample_Location:Doveton"

compute_core_microbiome.py -i /data/rebekah_data/bioms/run4_5/closed/split/otu_table_Penny_doubleton_filtered.biom -o /data/rebekah_data/bat_study/core_microbiomes/re_run_yarra/ --mapping_fp /data/rebekah_data/bioms/run4_5/closed/split/mapping_Penny.txt --valid_states "Sample_Location:Yarra_Bend"

- Comparison of beta diversity

compare_categories.py --method permanova -i /data/rebekah_data/bat_study/core_diversity/bdiv_even40000/weighted_unifrac_dm.txt -m /data/rebekah_data/bioms/run4_5/closed/split/mapping_Penny.txt -c Sample_Location -n 999 -o /data/rebekah_data/bat_study/core_diversity/bdiv_even40000/stats

compare_categories.py --method anosim -i /data/rebekah_data/bat_study/core_diversity/bdiv_even40000/weighted_unifrac_dm.txt -m /data/rebekah_data/bioms/run4_5/closed/split/mapping_Penny.txt -c Sample_Location -n 999 -o /data/rebekah_data/bat_study/core_diversity/bdiv_even40000/stats

- Pathogen BLAST table analysis comparison to GreenGenes

split_otu_table.py -i /data/rebekah_data/bioms/run4_5/closed_BLAST/original/otu_table.biom -o /data/rebekah_data/bioms/run4_5/closed_BLAST/split -m /data/rebekah_data/bat_study/mapping/mapping4_5_Yarra_Doveton_bat.txt -f Project

biom summarize-table -i /data/rebekah_data/bioms/run4_5/closed_BLAST/split/otu_table_Penny.biom -o /data/rebekah_data/bioms/run4_5/closed_BLAST/split/otu_table_Penny_summary.txt

filter_otus_from_otu_table.py -i /data/rebekah_data/bioms/run4_5/closed_BLAST/split/otu_table_Penny.biom -o /data/rebekah_data/bioms/run4_5/closed_BLAST/split/otu_table_Penny_singletons.biom -n 2

biom summarize-table -i /data/rebekah_data/bioms/run4_5/closed_BLAST/split/otu_table_Penny_singletons.biom -o /data/rebekah_data/bioms/run4_5/closed_BLAST/split/otu_table_Penny_singletons_summary.txt

filter_otus_from_otu_table.py -i /data/rebekah_data/bioms/run4_5/closed_BLAST/split/otu_table_Penny_singletons.biom -o /data/rebekah_data/bioms/run4_5/closed_BLAST/split/otu_table_Penny_doubletons.biom -n 3

biom summarize-table -i /data/rebekah_data/bioms/run4_5/closed_BLAST/split/otu_table_Penny_doubletons.biom -o /data/rebekah_data/bioms/run4_5/closed_BLAST/split/otu_table_Penny_doubletons_summary.txt

plot_taxa_summary.py -i /data/rebekah_data/bioms/run4_5/closed_BLAST/otu_table_converted_bat_only_taxa_n10.txt -o /data/rebekah_data/bat_study/taxa_summaries/blast_n10_plots -c bar

- - See above for the generation of this .txt file; this was just a tidy up
- Generated species level comparative figure for GreenGenes discussion (have tidies and compiled the results from greengenes and blast into single file to ensure coloration is the same)

filter_otus_from_otu_table.py -i /data/rebekah_data/bioms/run4_5/closed/split/otu_table_Penny.biom -o /data/rebekah_data/bioms/run4_5/closed/split/otu_table_Penny_n10.biom -n 11

plot_taxa_summary.py -i /data/rebekah_data/bat_study/taxa_summaries/closed_n10/otu_table_converted_bat_only_taxa_n10_blast.txt -o data/rebekah_data/bat_study/taxa_summaries/closed_n10/plots_combined/ -c bar

### 22/11/16

- All animal diversity analysis

split_otu_table.py -i /data/rebekah_data/bioms/run4_5/closed/4_5_no_singlteons.biom -o /data/rebekah_data/bioms/run4_5/closed/split/animals -m /data/rebekah_data/bat_study/mapping/mapping4_5_animal_human_faeces.txt -f Retain

core_diversity_analyses.py -i /data/rebekah_data/bioms/run4_5/closed/split/animals/4_5_no_singlteons_Yes.biom -p /data/rebekah_data/bioms/run4_5/closed/split/bats/metrics.txt -m /data/rebekah_data/bioms/run4_5/closed/split/animals/mapping_Yes.txt -e 20000 -t /data/rebekah_data/Yarra_Data/bioms/closed/pre-merged/97_otus.tree -o /data/rebekah_data/bat_study/core_diversity_animals

- Alpha diversity comparison for Stdev and P value based on Sample_Type
  - Was completed for each metric

compare_alpha_diversity.py -i /data/rebekah_data/bat_study/core_diversity_animals/arare_max20000/alpha_div_collated/simpson_e.txt -o /data/rebekah_data/bat_study/core_diversity_animals/arare_max20000/alpha_div_collated/simpson_e_compare.txt -p fdr -n 1000 -c Sample_Type -m /data/rebekah_data/bioms/run4_5/closed/split/animals/mapping_Yes.txt
