# Supplementary material for: What’s the risk? Identifying potential human pathogens within grey-headed flying foxes faeces
Source: PLoS One. 2018 Jan 23;13(1):e0191301. doi: 10.1371/journal.pone.0191301 (PMC5779653; doi:10.1371/journal.pone.0191301)
Supplement: S5 File — Taxonomic legend for Fig 4. (PDF) [file pone.0191301.s005.pdf]

Shigella_flexneri
Haemophilus_sputorum
Buttiauxella_ferragutiae
Acinetobacter_lwoffii
Citrobacter_amalonaticus
Acinetobacter_variabilis
Yersinia_rohdei
Escherichia_marmotae
Klebsiella_pneumoniae
Serratia_odorifera
Rahnella_aquatilis
Acinetobacter_kyonggiensis
Sphingobacterium_faecium
Pseudomonas_marincola
Massilia_dura
Turicibacter_sanguinis
Serratia_ficaria
Klebsiella_singaporensis
Enterobacter_hormaechei
Erwinia_iniecta
Pantoea_gaviniae
Pseudomonas_moraviensis
Sphingobacterium_kitahiroshimense
Pantoea_allii
Yersinia_enterocolitica
Actinobacillus_minor
Cedecea_neteri
Rosenbergiella_nectarea
Acinetobacter_radioresistens
Pseudomonas_amygdali
Ewingella_americana
Acinetobacter_johnsonii
Pseudomonas_taetrolens
Acinetobacter_boissieri
Streptococcus_salivarius
Escherichia_coli
Salmonella_enterica_subsp._arizonae
Serratia_fonticola
Pantoea_vagans
Pantoea_calida
Yersinia_massiliensis
Budvicia_aquatica
Duganella_zoogloeoides
Escherichia_vulneris
Massilia_alkalitolerans
Yersinia_enterocolitica_subsp._palearctica
Massilia_jejuensis
Acinetobacter_junii
Massilia_plicata
Acinetobacter_gandensis
Tatumella_terrea
Pseudomonas_helmanticensis
Acinetobacter_haemolyticus
Clostridium_celatum
Pseudomonas_flavescens
Shigella_dysenteriae
Acinetobacter_brisouii
Flavobacterium_granuli
Massilia_haematophila
Streptococcus_suis
Pseudomonas_graminis
Pedobacter_trunci
Chryseobacterium_scophthalmum
Massilia_albidiflava
Erwinia_typographi
[Curtobacterium]_plantarum
Serratia_grimesii
Flavobacterium_ginsengisoli
Cronobacter_condimenti
Duganella_phyllosphaerae
Cronobacter_sakazakii
Streptococcus_sanguinis
Clostridium_puniceum
Pseudomonas_straminea
Clostridium_chromiireducens
Lelliottia_amnigena
Pedobacter_roseus
Cronobacter_muytjensii
Epilithonimonas_tenax
Pantoea_brenneri
Kluyvera_intermedia
Pantoea_agglomerans
Cronobacter_turicensis
Chryseobacterium_aquaticum
Erwinia_billingiae
Raoultella_planticola
Chryseobacterium_solani
Yersinia_kristensenii
Ursidibacter_arcticus
Comamonas_koreensis
Gibbsiella_dentisursi
Erwinia_persicina
Pantoea_conspicua
Pseudomonas_japonica
Acinetobacter_indicus
Comamonas_terrigena
Yersinia_pseudotuberculosis
Stenotrophomonas_rhizophila
Shigella_boydii
Telluria_mixta
Raoultella_electrica
Luteibacter_rhizovicinus
Curvibacter_fontanus
Pseudomonas_cichorii
Flavobacterium_compostarboris
Pantoea_anthophila
Flavobacterium_chungangense
Pantoea_eucalypti
Cronobacter_zurichensis
Cronobacter_pulveris
Sphingomonas_ginsengisoli'_Hoang_et_al._2012
Enterobacter_soli
Pedobacter_hartonius
Chryseobacterium_soldanellicola
Plesiomonas_shigelloides
Vespertiliibacter_pulmonis
Variovorax_boronicumulans
Pedobacter_ginsengiterrae
Pedobacter_borealis
Pseudomonas_lutea
Massilia_aurea
Kluyvera_georgiana
Pantoea_wallisii
Acinetobacter_venetianus
Pedobacter_ginsengisoli
Acinetobacter_baylyi
Acinetobacter_puyangensis
Flavobacterium_spartansii
Enterobacter_asburiae
Pectobacterium_carotovorum_subsp._brasiliense
Serratia_quinivorans
Massilia_brevitalea
Pseudomonas_koreensis
Pseudomonas_savastanoi
Cetobacterium_somerae
Flavobacterium_pectinovorum
Epilithonimonas_lactis
Pseudomonas_vranovensis
Saccharibacillus_sacchari
Actinobacillus_porcinus
Streptococcus_porcorum
Rhizobium_pusense
Skermanella_aerolata
Flavobacterium_oncorhynchi
Escherichia_albertii
Pseudomonas_syringae_pv._tomato_str._DC3000
Massilia_varians
Rouxiella_chamberiensis
Cronobacter_dublinensis_subsp._lactaridi
Acinetobacter_bouvetii
Acidovorax_radicis
Acinetobacter_qingfengensis
Pseudomonas_rhodesiae
Sphingomonas_jinjuensis
Arthrobacter_enclensis
Haemophilus_parainfluenzae
Clostridium_perfringens
Stenotrophomonas_chelatiphaga
Acinetobacter_baumannii
Massilia_yuzhufengensis
Paenibacillus_nicotianae
Lactococcus_lactis_subsp._cremoris
Duganella_sacchari
Raoultella_ornithinolytica
Sphingopyxis_witflariensis
Novosphingobium_barchaimii
Gibbsiella_papilionis
Pseudomonas_migulae
Kluyvera_ascorbata
Pseudomonas_brassicacearum
Pedobacter_suwonensis
Pseudomonas_viridiflava
Pantoea_ananatis
Streptococcus_phocae
Pseudomonas_lundensis
Erwinia_toletana
Janibacter_hoylei
Acinetobacter_guillouiae
Dyadobacter_hamtensis
Klebsiella_oxytoca
Acinetobacter_guangdongensis
Citrobacter_murliniae
Flavobacterium_qiangtangense
Flavobacterium_saccharophilum
Comamonas_terrae
Comamonas_kerstersii
Dyadobacter_psychrophilus
Acinetobacter_schindleri
Massilia_aerilata
Flavobacterium_cutihirudinis
Flavobacterium_fryxellicola
Actinobacillus_equuli_subsp._haemolyticus
Erwinia_aphidicola
Pseudomonas_kuykendallii
Flavobacterium_gyeonganense
Klebsiella_variicola
Alkanindiges_illinoisensis
Pseudomonas_mosselii
Kluyvera_cryocrescens
Duganella_radicis
Pedobacter_soli
Cronobacter_helveticus
Pseudoxanthomonas_spadix
Saccharibacillus_kuerlensis
Chryseobacterium_soli
Flavobacterium_glaciei
Pedobacter_duraquae
Pseudomonas_fragi
Polaromonas_vacuolata
Erwinia_mallotivora
Chryseobacterium_piscicola
Pedobacter_koreensis
Pseudomonas_plecoglossicida
Rheinheimera_soli
Altererythrobacter_indicus
Pseudomonas_frederiksbergensis
Massilia_lurida
Pseudomonas_caricapapayae
Pseudomonas_congelans
Novosphingobium_arabidopsis
Clostridium_disporicum
Enterococcus_sulfureus
Enterococcus_malodoratus
Obesumbacterium_proteus
Acidovorax_soli
Pantoea_septica
Pseudomonas_reinekei
Pseudomonas_chlororaphis_subsp._aureofaciens
Enterococcus_casseliflavus
Serratia_plymuthica
Novosphingobium_lindaniclasticum
Asanoa_hainanensis
Pedobacter_panaciterrae
Pseudomonas_libanensis
Pseudomonas_coleopterorum
Dyadobacter_ginsengisoli
Limnohabitans_planktonicus
Cellulomonas_iranensis
Citrobacter_youngae
Kingella_kingae
Friedmanniella_antarctica
Paenibacillus_anaericanus
Enterococcus_saccharolyticus
Flavobacterium_banpakuense
Chryseobacterium_ginsengisoli
Pectobacterium_carotovorum
Paenibacillus_taohuashanense
Cronobacter_dublinensis_subsp._lausannensis
Citrobacter_koseri
Novosphingobium_pentaromativorans
Acinetobacter_gerneri
Aggregatibacter_aphrophilus
Tatumella_saanichensis
Flavobacterium_nitratireducens
Modestobacter_versicolor
Massilia_niabensis
Pseudomonas_deceptionensis
Streptococcus_sanguinis_SK1
Pantoea_intestinalis
Pseudomonas_indica
Sphingomonas_yunnanensis
Gibbsiella_greigii
Pedobacter_agri
Pseudomonas_protegens
Pseudomonas_brenneri
Pantoea_dispersa
Buttiauxella_gaviniae
Erwinia_oleae
Hafnia_psychrotolerans
Brevundimonas_staleyi
Massilia_consociata
Brevundimonas_bacteroides
Staphylococcus_nepalensis
Variovorax_ginsengisoli
Neisseria_weaveri
Arthrobacter_psychrochitiniphilus
Paenibacillus_borealis
Dyadobacter_arcticus
Alkanindiges_hongkongensis
Cronobacter_universalis
Hymenobacter_norwichensis
Sanguibacter_keddieii
Streptococcus_peroris
Falsirhodobacter_halotolerans
Pseudomonas_kilonensis
Caenimonas_terrae
Roseomonas_vinacea
Shinella_kummerowiae
Acinetobacter_tjernbergiae
Sphingobacterium_composti_Ten_et_al._2007
Serratia_entomophila
Sphingomonas_astaxanthinifaciens
Flavobacterium_rivuli
Enterobacter_ludwigii
Hymenobacter_gelipurpurascens
Massilia_eurypsychrophila
Hymenobacter_ginsengisoli
Acinetobacter_gyllenbergii
Noviherbaspirillum_suwonense
Pseudomonas_brassicacearum_subsp._neoaurantiaca
Erwinia_tasmaniensis
Pseudomonas_saponiphila
Rosenbergiella_australoborealis
Enterobacter_aerogenes
Rhizobacter_gummiphilus
Pedobacter_westerhofensis
Dyadobacter_koreensis
Lonsdalea_quercina_subsp._britannica
Pseudomonas_abietaniphila
Ottowia_thiooxydans
Pedobacter_xixiisoli
Clostridium_diolis
Sphingomonas_cynarae
Rugamonas_rubra
Ramlibacter_ginsenosidimutans
Lonsdalea_quercina_subsp._iberica
Methylobacterium_radiotolerans
Flavobacterium_seoulense
Chitinophaga_sancti
Massilia_namucuonensis
Sphingobacterium_nematocida
Haemophilus_pittmaniae
Arcticibacter_pallidicorallinus
Sphingobacterium_alimentarium
Pantoea_deleyi
Chryseobacterium_xinjiangense
Xenophilus_arseniciresistens
Pseudomonas_putida
Brevundimonas_terrae
Providencia_alcalifaciens
Pedobacter_namyangjuensis
Cellulomonas_hominis
Sphingomonas_aurantiaca
Moellerella_wisconsensis
Flavobacterium_hauense
Pseudorhodoferax_soli
Acinetobacter_antiviralis
Tatumella_citrea
Pedobacter_jejuensis
Massilia_suwonensis
Hafnia_paralvei
Variovorax_dokdonensis
Sphingobium_sufflavum
Blastococcus_aggregatus
Sphingomonas_wittichii
Cellvibrio_fibrivorans
Dyadobacter_alkalitolerans
Phaseolibacter_flectens
Roseateles_aquatilis
Citrobacter_gillenii
Rhodanobacter_umsongensis
Pedobacter_ginsenosidimutans
Pluralibacter_pyrinus
Erwinia_papayae
Tardiphaga_robiniae
Sphingomonas_oligophenolica
Pantoea_theicola
Pseudomonas_costantinii
Sphingobium_endophyticum
Actinobacillus_indolicus
Pseudomonas_tolaasii
Flavobacterium_frigidimaris
Arcticibacter_svalbardensis
Rhizobium_ecuadorense
Noviherbaspirillum_canariense
Serratia_marcescens_subsp._sakuensis
Gemella_parahaemolysans
Flavobacterium_xueshanense
Pantoea_stewartii_subsp._indologenes
Hymenobacter_tibetensis
Tatumella_ptyseos
Polaromonas_naphthalenivorans
Samsonia_erythrinae
Rhodococcus_qingshengii
Variovorax_defluvii
Haemophilus_parahaemolyticus
Pseudomonas_endophytica
Buttiauxella_brennerae
Pseudomonas_cuatrocienegasensis
Pseudomonas_extremorientalis
Pseudomonas_mandelii
Flavobacterium_chilense
Flavobacterium_terrigena
Rhizobium_soli
Delftia_litopenaei
Pseudomonas_azotoformans
Flavobacterium_daejeonense
Pedobacter_aquatilis
Massilia_tieshanensis
Escherichia_hermannii
Pseudomonas_chlororaphis_subsp._piscium
Enterobacter_kobei
Fusobacterium_nucleatum_subsp._polymorphum
Ramlibacter_solisilvae
Modestobacter_marinus
Arthrobacter_chlorophenolicus
Rhodopseudomonas_pentothenatexigens
Corynebacterium_ilicis
Hoeflea_olei
Flavobacterium_glycines
Micromonospora_eburnea
Neisseria_elongata_subsp._nitroreducens
Pseudomonas_taeanensis
Chryseobacterium_indoltheticum
Acinetobacter_soli
Flavobacterium_succinicans
Streptococcus_porcinus
Chryseobacterium_jeonii
Rosenbergiella_epipactidis
Sphingobium_qiguonii
Caulobacter_profundus
Polaromonas_aquatica
Novosphingobium_ginsenosidimutans
Flavobacterium_noncentrifugens
Kineococcus_aurantiacus
Sphingomonas_glacialis
Carnobacterium_viridans
Belnapia_soli
Massilia_arvi
Kineosporia_mesophila
Hymenobacter_soli
Dyadobacter_sediminis
Flavobacterium_subsaxonicum
Thermomonas_carbonis
Actinoplanes_ianthinogenes
Neisseria_elongata
Pseudomonas_palleroniana
Pseudomonas_asturiensis
Dyadobacter_crusticola
Yersinia_aldovae
Aquabacterium_commune
Stenotrophomonas_terrae
Spirosoma_fluviale
Flavobacterium_myungsuense
Variovorax_soli
Pseudomonas_seleniipraecipitans
Pseudoclavibacter_faecalis
Leptothrix_mobilis
Yersinia_ruckeri
Brevundimonas_lenta
Brevundimonas_bullata
Massilia_kyonggiensis
Pseudomonas_thivervalensis
Novosphingobium_soli
Sphingopyxis_chilensis
Humicoccus_flavidus
Erwinia_amylovora
Sphingomonas_phyllosphaerae
Pedobacter_ureilyticus
Pseudomonas_soli
Rhodobacter_sphaeroides
Psychrobacter_alimentarius
Enterococcus_haemoperoxidus
Paucibacter_toxinivorans
Sphingobacterium_yanglingense
Agrobacterium_larrymoorei
Microbacterium_enclense
Pedobacter_cryoconitis
Pseudomonas_alcaliphila
Hymenobacter_psychrotolerans
Methylobacterium_bullatum
Paenibacillus_wynnii
Delftia_acidovorans
Flavobacterium_reichenbachii
Salmonella_bongori
Raoultella_terrigena
Flavobacterium_defluvii
Citrobacter_freundii
Pelomonas_aquatica
Flavobacterium_limicola
Flavobacterium_hercynium
Streptococcus_thermophilus
Pedobacter_lotistagni
Paracoccus_niistensis
Rhizobacter_dauci
Pedobacter_insulae
Sphingomonas_aerophila
Haemophilus_haemoglobinophilus
Spirosoma_endophyticum
Pantoea_rodasii
Caulobacter_henricii
Sphingomonas_mali
Pseudomonas_vancouverensis
Pseudomonas_parafulva
Salmonella_enterica_subsp._houtenae
Epilithonimonas_xixisoli
Flavobacterium_aquidurense
Serratia_rubidaea
Acidovorax_facilis
Cellvibrio_ostraviensis
Flavobacterium_yonginense
Dyadobacter_fermentans
Paraherbaspirillum_soli
Pedobacter_steynii
Novosphingobium_chloroacetimidivorans
Rhizobium_galegae
Actinobacillus_lignieresii
Roseomonas_musae
Devosia_crocina
Massilia_timonae
Pedobacter_oryzae
Sphingomonas_insulae
Leifsonia_bigeumensis
Rhodoferax_saidenbachensis
Flavobacterium_resistens
Chryseobacterium_yonginense
Pseudomonas_lini
Phenylobacterium_haematophilum
Pseudomonas_syringae
Pseudomonas_rhizosphaerae
Pedobacter_piscium
Variovorax_guangxiensis
Acidovorax_konjaci
Citrobacter_sedlakii
Pseudomonas_mediterranea
Yersinia_intermedia
Bifidobacterium_adolescentis
Chryseobacterium_ginsenosidimutans
Terrimonas_arctica
Acinetobacter_kookii
Sphingobium_chlorophenolicum
Janthinobacterium_agaricidamnosum
Mucilaginibacter_ximonensis
Aquabacterium_citratiphilum
[Clostridium]_hiranonis
Pseudomonas_alcaligenes
Paracoccus_limosus
Citrobacter_werkmanii
Yersinia_pekkanenii
Acinetobacter_beijerinckii
Pseudocitrobacter_faecalis
Ramlibacter_henchirensis
Devosia_lucknowensis
Chryseobacterium_formosense
Hymenobacter_algoricola
Sphingobium_aromaticiconvertens
Yersinia_similis
Pedobacter_yonginense
Curvibacter_delicatus
Klebsiella_pneumoniae_subsp._ozaenae
Rhodoferax_antarcticus
Serratia_nematodiphila
Nocardioides_daedukensis
Lautropia_mirabilis
Pseudomonas_marginalis
Bradyrhizobium_canariense
Curtobacterium_herbarum
Marmoricola_aquaticus
Pelosinus_propionicus
Tatumella_punctata
Acinetobacter_bereziniae
Pseudoxanthomonas_sacheonensis
Luteolibacter_pohnpeiensis
Kosakonia_cowanii
Pelomonas_saccharophila
Arsenicicoccus_bolidensis
Sphingobium_yanoikuyae
Brevundimonas_variabilis
Salmonella_subterranea
Qipengyuania_sediminis
Noviherbaspirillum_malthae
Budvicia_diplopodorum
Pluralibacter_gergoviae
Devosia_insulae
Sphingomonas_naasensis
Avibacterium_endocarditidis
Luteimonas_abyssi
Pseudomonas_guineae
Haemophilus_haemolyticus
Kosakonia_pseudosacchari
Acinetobacter_towneri
Rhizobium_cellulosilyticum
Flavobacterium_denitrificans
Paenibacillus_sonchi
Aquabacterium_fontiphilum
Arthrobacter_niigatensis
Roseomonas_aerilata
Variovorax_paradoxus
Citrobacter_rodentium
Flavobacterium_omnivorum
Serratia_ureilytica
Flavobacterium_tructae
Mycobacterium_hodleri
Serratia_liquefaciens
Flavobacterium_piscis
Pelomonas_puraquae
Pseudomonas_cremoricolorata
Actinoplanes_consettensis
Sphingobium_francense
Pseudomonas_fluorescens
Chryseobacterium_chaponense
Yokenella_regensburgei
Flavobacterium_arsenitoxidans
Acinetobacter_nectaris
Sphingobium_vermicomposti
Duganella_violaceinigra
Yersinia_bercovieri
Kocuria_assamensis
Delftia_tsuruhatensis
Rhodococcus_coprophilus
Hymenobacter_metalli
Pseudomonas_batumici
Pseudomonas_mendocina
Ralstonia_insidiosa
Flavobacterium_caeni
Mucilaginibacter_myungsuensis
Roseomonas_aerophila
Cellvibrio_gandavensis
Shinella_fusca
Roseomonas_aquatica
Saxeibacter_lacteus
Clostridium_magnum
Chitinophaga_oryziterrae
Luteolibacter_arcticus
Luteolibacter_luojiensis
Tersicoccus_phoenicis
Brevundimonas_intermedia
Erwinia_rhapontici
Subdoligranulum_variabile
Pseudorhodoferax_aquiterrae
Psychrobacter_luti
Psychrobacter_pulmonis
Rhizobium_huautlense
Flavobacterium_hydatis
Pedobacter_heparinus
Enterococcus_canintestini
Extensimonas_vulgaris
Williamsia_faeni
Polynucleobacter_necessarius_subsp._asymbioticus
Mucilaginibacter_lutimaris
Pseudomonas_sabulinigri
Mesorhizobium_australicum
Streptococcus_lutetiensis
Mucilaginibacter_rigui
Enterococcus_canis
Blastocatella_fastidiosa
Yimella_lutea
Sphingomonas_desiccabilis
Brachybacterium_squillarum
Mitsuaria_chitosanitabida
Pseudochrobactrum_saccharolyticum
Pseudoxanthomonas_dokdonensis
Beijerinckia_indica_subsp._indica
Pantoea_stewartii
Sphingomonas_vulcanisoli
Microvirga_guangxiensis
Flavobacterium_flevense
Enterococcus_mundtii
Cryptosporangium_minutisporangium
Cronobacter_malonaticus
Citrobacter_braakii
Dermacoccus_abyssi
Rhizobacter_fulvus
Sphingobacterium_canadense
Sphingomonas_soli
Erwinia_piriflorinigrans
Enterobacter_cloacae
Serratia_marcescens
Pseudomonas_synxantha
Actinoplanes_lutulentus
Blastococcus_endophyticus
Flavitalea_populi
Pseudomonas_panacis
Arthrobacter_nicotinovorans
Brenneria_salicis
Cellulomonas_pakistanensis
Acidovorax_delafieldii
Sinorhizobium_meliloti
Vasilyevaea_mishustinii
Arcobacter_cryaerophilus
Rhizobium_mongolense
Pseudorhodoferax_caeni
Cellulomonas_massiliensis
Azohydromonas_australica
Oxalicibacterium_faecigallinarum
Knoellia_flava
Pseudomonas_tuomuerensis
Neisseria_animalis
Pseudomonas_moorei
Cellvibrio_vulgaris
Serratia_symbiotica
Curtobacterium_flaccumfaciens
Kosakonia_sacchari
Streptococcus_macedonicus
Pseudonocardia_hydrocarbonoxydans
Sphingobium_fuliginis
Youhaiella_tibetensis
Polaromonas_jejuensis
Pseudomonas_chlororaphis_subsp._aurantiaca
Mycobacterium_psychrotolerans
Rheinheimera_nanhaiensis
Friedmanniella_lacustris
Hymenobacter_flocculans
Rhizobium_rosettiformans
Afipia_broomeae
Micromonospora_coerulea
Spirosoma_rigui
Pseudomonas_azotifigens
Erwinia_psidii
Flavobacterium_macrobrachii
Xanthomonas_vasicola
Alsobacter_metallidurans
Rhodococcus_fascians
Microvirga_aerilata
Actinoplanes_friuliensis
Acidovorax_defluvii
Devosia_yakushimensis
Marmoricola_korecus
Chryseobacterium_polytrichastri
Cosenzaea_myxofaciens
Xanthomonas_maliensis
Gemella_haemolysans
Actinoplanes_globisporus
Pedobacter_nutrimenti
Trabulsiella_guamensis
Methylobacterium_cerastii
Adhaeribacter_terreus
Klebsiella_pneumoniae_subsp._rhinoscleromatis
Kineococcus_xinjiangensis
Flavobacterium_fluvii
Flavobacterium_ginsenosidimutans
Caulobacter_vibrioides
Serratia_fonticola_RB-25
Arthrobacter_gandavensis
Pantoea_cypripedii
Janibacter_cremeus
Streptococcus_vestibularis
Cellulomonas_cellasea
Novosphingobium_tardaugens
Kocuria_aegyptia
Segetibacter_koreensis
Novosphingobium_hassiacum
Janthinobacterium_lividum
Morganella_morganii_subsp._sibonii
Stenotrophomonas_pavanii
Renibacterium_salmoninarum
Streptococcus_cristatus
Pseudomonas_monteilii
Hymenobacter_fastidiosus
Oerskovia_paurometabola
Piscinibacter_aquaticus
Leuconostoc_lactis
Modestobacter_roseus
Roseateles_terrae
Yersinia_entomophaga
Rhizobium_herbae
Microbacterium_rhizomatis
Phytohabitans_flavus
Rhodobacter_ovatus
Flavobacterium_koreense
Burkholderia_humi_Vandamme_et_al._2013
Kaistia_defluvii
Sphingomonas_aquatilis
Dyadobacter_tibetensis
Altererythrobacter_troitsensis
Vibrio_litoralis
Kinneretia_asaccharophila
Pedobacter_tournemirensis
Luteolibacter_yonseiensis
Mucilaginibacter_mallensis
Xenophilus_aerolatus
Rubrivivax_gelatinosus
Microvirga_flocculans
Nesterenkonia_flava
Pedobacter_caeni
Oxalicibacterium_solurbis
Acinetobacter_seohaensis
[Clostridium]_sordellii
Pseudomonas_stutzeri
Zhizhongheella_caldifontis
Algoriphagus_jejuensis
Phenylobacterium_muchangponense
Enterococcus_dispar
Aureimonas_ureilytica
Dactylosporangium_thailandense
Brevundimonas_alba
Enterococcus_gallinarum
Pseudoclavibacter_bifida
Chitinibacter_suncheonensis
Herminiimonas_fonticola
Sphingomonas_hankookensis
Pseudomonas_cedrina
Brevundimonas_vesicularis
Chryseobacterium_aahli
Flavisolibacter_ginsengiterrae
Sphingobium_limneticum
Bradyrhizobium_oligotrophicum
Chryseobacterium_shigense
Kingella_denitrificans
Acidovorax_wautersii
Sphingomonas_xinjiangensis
Blastococcus_jejuensis
Arthrobacter_ureafaciens
Cellulomonas_xylanilytica
Sediminihabitans_luteus
Intrasporangium_calvum
Knoellia_subterranea
Arthrobacter_pascens
Arthrobacter_psychrolactophilus
Phytohabitans_suffuscus
Mycobacterium_vaccae
Sphingobacterium_multivorum
Staphylococcus_epidermidis
Brevundimonas_diminuta
Brevundimonas_poindexterae
Methylobacterium_adhaesivum
Agrobacterium_vitis
Carludovica_palmata
Lupinus_luteus
Nelumbo_nucifera
Oenothera_berteroana
Sarcandra_grandifolia
Zea_luxurians
Sphingomonas_asaccharolytica
Sphingomonas_echinoides
Acidovorax_wohlfahrtii
Massilia_aerolata
Methylotenera_mobilis
Aeromonas_hydrophila
Enterobacter_cowanii
Enterobacter_gergoviae
Enterobacter_pyrinus
Enterobacter_turicensis
Erwinia_dispersa
Erwinia_soli
Salmonella_enterica
Shigella_sonnei
Trabulsiella_farmeri
Xenorhabdus_bovienii
Pseudomonas_umsongensis
Pseudomonas_veronii
Stenotrophomonas_acidaminiphila
Stenotrophomonas_maltophilia
